# Multi-Omics Analysis Demonstrates the Critical Role of Non-Ethanolic Components of Alcoholic Beverages in the Host Microbiome and Metabolome: A Human- and Animal-Based Study

**DOI:** 10.3390/microorganisms11061501

**Published:** 2023-06-05

**Authors:** Priyanka Sarkar, Raghuram Kandimalla, Anupam Bhattacharya, Romi Wahengbam, Madhusmita Dehingia, Mohan Chandra Kalita, Narayan Chandra Talukdar, Rupjyoti Talukdar, Mojibur R. Khan

**Affiliations:** 1 Molecular Biology and Microbial Biotechnology Laboratory, Life Science Division, Institute of Advanced Study in Science and Technology (IASST), Department of Science and Technology, Government of India, Paschim Boragaon, Garchuk, Guwahati 781035, Assam, India; 2 Wellcome/DBT (Indian Alliance) Lab, Institute of Translational Research, Asian Healthcare Foundation, Asian Institute of Gastroenterology (AIG Hospitals), Hyderabad 500032, Telangana, India; 3 Brown Cancer Centre, University of Louisville, Louisville, KY 40202, USA; 4 Centre for Infectious Diseases, Biological Sciences and Technology Division, CSIR-North East Institute of Science and Technology, Jorhat 785006, Assam, India; 5 Department of Biotechnology, Gauhati University, Guwahati 781014, Assam, India; 6 Faculty of Science, Assam Down Town University, Panikhaiti, Guwahati 781026, Assam, India

**Keywords:** gut microbiome, alcoholic beverages, next-generation sequencing, fecal and blood metabolomics, GC-MS/MS

## Abstract

It is known that alcoholic beverages alter the human gut microbiome. This study focused on the potential impact of non-ethanolic ingredients in whisky on the gut bacteriome. A pilot study was carried out on 15 whisky drinkers, 5 rice beer drinkers, and 9 non-drinkers to determine the effect of alcoholic beverages on the host microbiome and metabolome. Additionally, a mouse model was used to assess the differential impact of three whisky brands (each with an equal ethanol concentration). The results indicate that the non-ethanolic components have an impact on the gut microbiome, as well as on the metabolites in blood and feces. The amount of *Prevotella copri*, a typical core Indian gut bacterium, decreased in both the human and mouse groups of whisky type 1, but an increase in abundance of Helicobacteriaceae (*p* = 0.01) was noticed in both groups. Additionally, the alcohol-treated cohorts had lower levels of short-chain fatty acids (SCFAs), specifically butyric acid, and higher amounts of lipids and stress marker IL1-ß than the untreated groups (*p* = 0.04–0.01). Furthermore, two compounds, ethanal/acetaldehyde (found in all the whisky samples) and arabitol (unique to whisky type 1), were tested in the mice. Similar to the human subjects, the whisky type 1 treated mouse cohort and the arabitol-treated group showed decreased levels of *Prevotella copri* (*p* = 0.01) in their gut. The results showed that non-ethanolic compounds have a significant impact on host gut bacterial diversity and metabolite composition, which has a further vital impact on host health. Our work further emphasizes the need to study the impact of non-ethanolic ingredients of alcoholic beverages on host health.

## 1. Introduction

Microbes in the gut derive energy from undigested luminal dietary residues and, in turn, participate in a plethora of metabolic functions and regulate the host physiology [1,2]. A state of dysbiosis occurs when the composition of the microbiota and its intrinsic metabolic interactions are altered, disrupting host–microbe homeostasis and thus further leading to a growing list of diseases [3,4,5]. Dietary habit is a major influencer of the gut microbiome [6]. Apart from a normal diet, alcoholic beverages have also been shown to influence gut microbiota, both positively and negatively. Recent reports have shown the possible linkage of long-term alcoholism with several diseases, such as colorectal cancer (CRC), alcoholic liver disease, etc. [3,4,5]. The consumption of alcoholic beverages for an extended period (>10 years) has also been correlated with detrimental gut bacterial dysbiosis [7]. On the contrary, polyphenols in beer have been observed to exert a positive impact on host health [8].

To the best of our knowledge, no study has been conducted to understand the effect of long-term consumption of whisky and its non-alcoholic components on the gut bacterial profile. The goal of this study was to investigate the effect of non-ethanolic compounds on host gut microbial and metabolite profiles.

## 2. Materials and Methods

### 2.1. Human Subjects, Fecal Sample Collection, and Preservation

This pilot-scale study on human subjects was approved by the Institutional Ethics Committee (Human Studies), Institute of Advanced Study in Science and Technology, Guwahati, India (application ID: IEC(HS)/IASST/1082/2014-15/7). This study was performed in accordance with relevant guidelines and regulations. Fecal samples were collected from volunteers who were long-term (12–22 years) drinkers of alcoholic beverages (7 drinkers of whisky type 1 (M), 5 drinkers of whisky types 2 and 3 (OB), 3 drinkers of whisky type 4 (R), 3 drinkers of rice beer (Rb), and 8 non-drinkers (C) from Guwahati and Bongaigaon (Assam, India)) after obtaining written informed consent. A standardized questionnaire was used to record a thorough history of their dietary, anthropometric, and health-related data. The inclusion criteria were the absence of antibiotics, probiotics, and other specific drug usages (which can alter the GI microbiome) for 6 months prior to the sample collection. Fecal samples were collected using RNAlater™ (Qiagen, Hilden, Germany) in sterile stool collection containers and stored at −80 °C until further analysis (Figure S1). Details of the drinking habits of the volunteers are provided in Table S1.

### 2.2. Study on the Effect of Alcoholic Beverages in Animal Model

A group of 8-week-old Swiss albino male mice were selected for this study. We first conducted a trial study by treating the mice with 250 µL of each alcoholic beverage mixed with sterile water (1:1, *v*/*v*) for 65 days to check any ethanol-induced toxicity or lethality in the animals. We collected fecal samples, performed DGGE-based gut bacterial analyses, and found heterogeneity in the results (Figure S2a). Hence, for the main experiment, we generated/bred F5 progeny of the same parental background to nullify the hosts’ genetic effects on the host microbiome (Figure S2b). After confirmation of homogeneity of the microbiome of the animals, we started the main experiments by dividing them into five treatment groups, which included three whisky groups {whisky type 1 (M), whisky type 2 (O), and whisky type 3 (B)); a group with 42.8% ethanol (E) (to analyze the effect of only ethanol (all the whisky types contained 42.8% ethanol)}; and a group without any treatment, serving as the control/untreated group (C). Each treatment group had 9 mice in 3 replicate cages to nullify the micro-environmental effects of the cage level. The mice were housed following a 12 h light/dark cycle, and whisky (250 µL mixed with sterile water, 1:1, *v*/*v*), 42.8% ethanol (250 µL mixed with sterile water, 1:1, *v*/*v*), and water (sterile, 250 µL) were fed via gavage according to their respective treatment group once daily for 60 days. The mice were kept on a standard rodent chow diet and drinking water. The mice’s body weights and dietary intakes per cage were tracked throughout the study. The volume of whisky to be fed was calculated based on the consumption habits of the human volunteers and their respective body weights ([9], Table S1). All of the experiments were conducted following the Committee for the Purpose of Control and Supervision of Experiments on Animals (CPCSEA) guidelines (registration number: 1706/GO/c/13/CPCSEA) and was approved by the Institutional Animal Ethical Committee of the Institute of Advanced Study in Science and Technology, Guwahati-35, Assam, India (IASST/IAEC/2014-15/1201).

### 2.3. DNA Extraction, PCR Amplification, and Denaturing Gradient Gel Electrophoresis (DGGE)

In the human study, fecal samples were collected at the time of enrolment. For the mouse study, fecal samples were collected twice daily using RNAlater™ (Qiagen, Germany) for the metagenomic analysis and using liquid N_2_ for the metabolomic study and were stored immediately at −80 °C. Metagenomic DNA was extracted as previously described [10]. The fecal metagenomic DNA of the mice was extracted every other day and processed for DGGE analysis to check for any microbiome differences in the groups.

PCR amplification of the V6–V8 region of 16S rDNA followed by DGGE analysis were performed in accordance with a previously described protocol [11]. Briefly, a DGGE analysis was performed in a 9% acrylamide/bisacrylamide (37.5:1) gel with a 35% to 60% denaturant gradient using an IngenyPhor U DGGE system (U-2, Ingeny, Amsterdam, The Netherland). A 100% denaturant contained 7M urea (Promega, Madison, WI, USA) and 40% deionized formamide (Sigma Aldrich, St. Louis, MO, USA). A 6% stacking gel was poured on top of the denaturing gel, and 600 ng of the PCR product was loaded into each well. A PCR-amplified product of *E. coli* 16S rDNA using the same primer pair was used as a control. A reference sample was developed by mixing a few samples with most of the representative bands. The electrophoresis was carried out at 70 V for 17 h at 60 °C in a 1X TAE buffer. The gel was visualized via silver staining using the modified version of a previously described protocol [12].

### 2.4. DGGE Band Sequencing

In the DGGE gel, each band was considered a bacterial ribotype, as coined previously by Tapia et al., 2014 [13]. Some of the bands could be eluted from the gel without losing their purity and, hence, sequenced directly, and for the remainder, cloning was performed. The bands were removed with a sterile, sharp scalpel, combined with 20 uL of a 1X TE buffer and then incubated at 37 °C for an overnight period. Quick centrifugation (12,000 rpm, 5 min at 4 °C) was performed to remove the supernatant, which was then employed as a template for PCR re-amplification and DGGE analysis. This process was repeated until the DGGE gel produced a single band. These samples were subjected to Sanger sequencing.

Cloning was performed with a reference sample for the generation of a clonal library. PCR products were first purified using the Gen-elute PCR purification kit (Sigma Aldrich, USA) and then cloned in *E. coli* JM109-competent cells using the pGEM-T Easy Vector (Promega, USA). Transformed *E. coli* cells were inoculated onto Luria-Bertani (LB) agar plates containing 100 ppm ampicillin, 80 ppm X-Gal, and 0.5 mM IPTG and incubated at 37 °C overnight. White transformed colonies were sub-cultured, and colony PCR was performed and subjected to DGGE analysis. Clones containing the band of interest were selected for plasmid DNA extraction and sequencing. Sequencing of the plasmid DNA was carried out in both directions, as described earlier. The DNA sequences were subjected to a BLAST analysis using the National Centre for Biotechnology Information (NCBI) blast resource (Table S2 and Figure S3) [14].

### 2.5. Next-Generation Sequencing (NGS) of Fecal 16S rDNA

Metagenomic DNA from 15 human fecal samples, which included 4 drinkers of whisky brand 1 (M), 3 drinkers of whisky brands 2 and 3 (OB), 3 drinkers of rice beer (Rb), and 5 non-drinkers (C), was subjected to next-generation sequencing (NGS) with Xcelris Genomics (Ahmedabad, India). In the mouse study, 30 samples were subjected to NGS analysis (3/group) from two study points: the 30th and 60th days of treatment. Since the mice were caged in 3 different cages (3 mice/cage) for each group, we pooled each cage sample into one, i.e., 50ng of extracted fecal metagenomic DNA from each mouse pooled into one sample per cage. The DNA was quantified using a Qubit dsDNA BR Assay kit (Thermo Fisher Scientific, Waltham, MA, USA). For the mouse studies, the fecal DNA profile of the 30th and 60th days of treatment was processed for NGS after observing the bacteriome differences based on the DGGE analysis. Bacterial diversity in the samples was analyzed using the V3-V4 region of 16S rDNA amplicon sequencing on the Illumina platform (MiSeq, 2 × 250 bp). Using a Nextera XT Index kit, 2 × 250 bp of MiSeq amplicon libraries were prepared (Illumina Inc., San Diego, CA, USA) following the Illumina16S metagenomic library preparation protocol (Part # 15044223 Rev. B). The primers V3 (forward, 5′CCTACGGGNGGCWGCAG3′) and V4 (reverse, 5′GACTACHVGGGTATCTAATCC3′) were used for the amplification. Furthermore, the amplicon libraries were purified using 1× Ampure XP beads, cross-checked on an Agilent DNA 1000 chip on bioanalyzer 2100, and quantified on a fluorometer using a Qubit dsDNA HS Assay kit (Life Technologies, Delhi, India); subsequently, 600 μL of 10 pM pooled libraries (mean peak size = ~600 to ~630 bp; spiked with 5% 12.5 pM PhiX Control) was then loaded on MiSeq for sequencing.

Barcode and sequencing primers were trimmed from the sequences. The raw NGS data obtained from the human and mouse studies were further analyzed using QIIME pipeline (v1.9.1) [15]. First, we assessed the quality score of the sequence using FastQC (https://www.bioinformatics.babraham.ac.uk/projects/fastqc/, 1 January 2017). Sequences with an average phred score lower than 25, containing ambiguous bases, with a homopolymer run exceeding 6, having mismatches in primers, or with a sequence length >100 bp were removed. DNA sequence reads with overlaps <10 bp and without any mismatch were assembled. We used the UCLUST method to cluster query sequences against a curated chimera-free 16S rRNA database (Greengenes v 13.8). A species-level operational taxonomic unit (OTU) was defined as the number of clusters sharing ≥97% sequence identity with Greengenes v.13.8. The OTUs without any taxonomic hit (at the species level) were further analyzed within the RDP database. For the α diversity analysis, the quality-filtered OTUs at the species level were rarefied at a phred quality score of 20 using the *multiple-rarefactions.py* script in QIIME pipeline. The β-diversity analysis with phylogenetic information of the gut microbial structures was analyzed using a PCoA (principal coordinate analysis) plot based on a UniFrac distance matrix. The sequence files have been submitted to SRA under the ID PRJNA931847.

### 2.6. Gas Chromatography–Mass Spectrometry (GC-MS)-Based Untargeted Metabolomic Profiling of Fecal and Plasma Samples

The fecal samples were first lyophilized for 24 h and then subjected to solvent extractions. Briefly, 50 mg of the fecal samples and 100 µL of the plasma samples were extracted using 200 µL of n-hexane and methanol (Merck, Darmstadt, Germany, Lichrosolve, 1:1, *v*/*v*) for 15 h at room temperature followed by centrifugation at 12,000 rpm at 4 °C for 5 min, and the supernatants were collected and pooled at equal quantities for the mice in the same cage. The samples were derivatized using N,O-Bis(trimethylsilyl)trifluoroacetamide (BSTFA) and N-Methyl-N-(trimethylsilyl) trifluoroacetamide (MSTFA) with pyridine (which acts as a catalyzer for silylation) to analyze the profiles. Furthermore, the supernatant was dried using a desiccator overnight and then subjected to derivatization according to a previously described protocol [9,16,17]. Briefly, methoximation was carried out by adding 40 µL of methoxyamine-HCl (20 mg/mL in pyridine), followed by incubation at 30 °C for 90 min. Then, trimethyl-silyl-based derivatization was performed using MSTFA (20 µL) and re-incubation was performed at 37 °C for 30 min.

### 2.7. Chemical Analysis of Alcoholic Beverages

We used a previously described approach to study the chemical fingerprints of the beverages. To check the volatile compounds, we first extracted the samples using n-hexane (Merck, Lichrosolve, 1:1, *v*/*v*) and then performed a GCMS analysis. Furthermore, we opted to use derivatization approaches to obtain better knowledge on the chemical fingerprints of the alcoholic beverages evaluated in this study. Sugars are key compounds in any alcoholic beverage. However, in the BSTFA-based analysis, the sugar molecules could not be detected, and therefore, a modified protocol using dichloromethane (DCM) and methanol extracts of the whisky samples followed by MSTFA-based derivatization was performed (we observed better profiles in terms of more compounds in the DCM extracts of the whisky samples, while the number of compounds were the same in both the DCM and methanol extracts of the fecal and plasma samples). Apart from these compounds, other non-ethanolic constituents of alcoholic beverages such as phenolics, aldehydes, lactones, etc. were also evaluated using both the BSTFA- and MSTFA-based approaches. An amount of 500 µL of the sample was extracted with methanol (Merck, Lichrosolve) at a 1:1 ratio, *v*/*v* for 15 h at room temperature. An amount of 200 µL of the supernatant was collected and dried in a desiccator overnight. All the dried extracts were processed according to a previously described protocol [16,17].

### 2.8. Programs for Gas Chromatography–Mass Spectrometry

A GC-MS analysis was performed with 1 µL of the prepared samples using a Shimadzu GC 2010 plus with triple quadruple MS (TP-8030) fitted with an EB-5MS column (length—30 m, thickness—0.25 µm, and ID—0.25 mm). For the BSTFA-based derivatized samples, the temperature program consisted of 60 °C for 2 min, was then raised to 250 °C at 4 °C/min, and was held for 15 min. The injection temperature was 250 °C, and the column flow rate was 1.0 mL/min, with He as a carrier gas. The mass spectrometer was operated in the electron ionization (EI) mode at 70 eV with an ion source temperature of 230 °C and a continuous scan from 45 to 800 m/z. The program for the MSTFA derivatized sample analysis was set up according to the program described earlier [17]. The peaks were identified by matching the mass spectra within the National Institute of Standards and Technology (NIST) library. The metabolite/compound peaks showing ≥85% similarity with the NIST database were considered for the downstream analysis. The peak area of each metabolite was considered based on the m/z ratio, and the peak intensities were considered for further analysis. Background noise (e.g., silanes/siloxanes) and solvent peaks were removed from the mass chromatograph of the samples. The compounds that were found repeatedly in at least three injections for all the replicates were considered. Furthermore, post-run system-generated metabolite data were recorded in Excel, and based on the peak area and retention time, the metabolites were segregated. A single compound showing multiple derivatized forms with a similar retention time (±0.05 min) was pooled into one for further analysis. The corresponding peak area (a peak area of zero was imputed for missing metabolites) was transformed into a relative percentage and considered for the downstream analysis.

### 2.9. Interleukin Assays

At the end of the experiment, the mice were sacrificed by cervical dislocation and blood samples were collected in K3-EDTA vials. The assays were performed with the plasma samples using the IL1β and TNFα mouse assay kits of R&D system (RESEARCH and Diagnostic Systems, Inc., Pearl River, LA, USA) according to the manufacturer’s instructions.

### 2.10. Statistical Analysis

As there are no prior analogous comparisons of the gut microbiota of different alcoholic beverage consumers, we decided to carry out the current comparison as a preliminary study. Therefore, no formal sample size calculation was performed for the human phase. For the mouse study, we kept 9 mice/treatment or group. The DGGE banding patterns were analyzed within the gel compare software (GelCompar II, v6.6; Applied Maths). Microbial richness/evenness (α diversity) was expressed as Chao 1, Shannon’s, Fisher’s, Observed OTU, and ACE indices based on the bacterial species level. Box and whisker plots were generated to represent the data, and Kruskal–Wallis test (KW) was performed for significance testing. ß diversity was evaluated via a principal-coordinate-analysis based on UniFrac distance matrix (both un-weighted and weighted) in QIIME v1.9.1, and a one-way permutational multivariate analysis of variance (PERMANOVA) using 10,000 permutations was carried out for the significance analysis. Per sample, rarefaction curves were constructed using the MicrobiomeAnalyst—based on the minimum library depth after library normalization to rule out any artefacts, following a previously described method [18]. The initial comparison among the microbes in all the groups was performed using the relative abundance of the taxa in each group.

The metabolites’ concentrations were normalized via log transformation and were subjected to a partial least squares discriminant analysis (PLS-DA), and the Kruskal–Wallis test or an analysis of variance (as appropriate) using MetaboAnalyst 5.0 [19]. A fold change analysis of the short-chain fatty acids (SCFAs) was also performed within MetaboAnalyst 5.0 [19]. For visualizations, we used MicrobiomeAnalyst, MetaboAnalyst, and ggplot2 package in R studio (v 4.2). The correlation between microbes and metabolites was measured using Spearman’s rank correlation in SPSS (v.23, IBM, Armonk, NY, USA) and then visualized using the Metscape plugin of Cytoscape (v. 3.8.2) [20,21].

## 3. Results

### 3.1. Effect of Alcoholic Beverages on the Human Gut Microbiome (Pilot Study)

**Figure 1:**
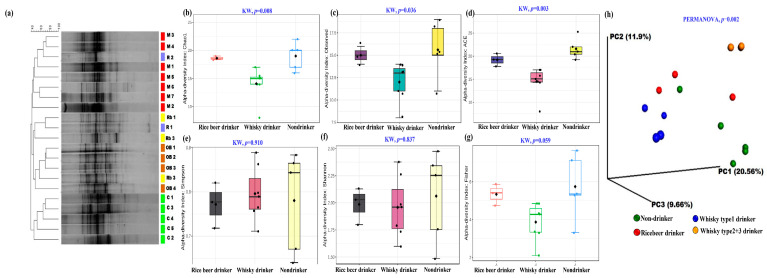
Gut bacterial profiles of the participants: (**a**) DGGE-based analysis shows the distinct clustering of the participants based on their preferred beverages. (**b**–**g**) α diversity indices showing the distinct differences in the microbiome of the drinkers vs. non-drinkers. (**h**) UniFrac distance (weighted)-based PCoA plot showing the microbial compositional differences among the groups. {code: C—control/non-drinker, M—whisky type 1 drinker, OB—whisky type 2 and 3 drinkers, and Rb—rice beer drinkers}.

Figure 1a depicts the DGGE analysis of the PCR-amplified 16S rDNA of fecal metagenomic DNA samples, revealing the differences in the gut bacteriome of the drinkers compared to the non-drinkers. A total of 44 ribotypes were detected in the DGGE gel, from which 16 were sequenced (Table S2 and Figure S3). Three ribotypes, ribotype 2 (*Eubacteria rectale*), ribotype 5 (*Clostridium* sp.), and ribotype 12 (*Succinovibrio dextrinosolvens*), were present in all volunteers (Figure S3). Of the remaining 14 ribotypes, ribotypes 1, 4, 7, and 8 (*Clostridium* sp.) were absent in the non-drinker group but were present in the drinkers, particularly at high intensity in the gut of type 1 whisky drinkers. Ribotypes 2, 6, 9, 11, and 16 (*Eubacteria rectale*) were either absent or present at very light intensities in the gut of the type 1 whisky drinkers. Furthermore, on average, significantly fewer ribotypes were detected in the gut of type 1 whisky drinkers compared with in the gut of non-drinkers (Figure S3).

We then performed a V3-V4-based NGS analysis to obtain a deeper understanding of the altered bacterial species in the groups and observed lower α diversity in the whisky cohorts (Figure 1b–g; species richness, Chao1, *p* = 0.008; species evenness, Fisher indices, *p* = 0.059). We also observed more prominent gut microbial differences between the whisky group 1 and group 2 drinkers, as depicted by the PCoA plot based on ß diversity (PERMANOVA, *p* = 0.001, Figure 1h).

**Figure 2:**
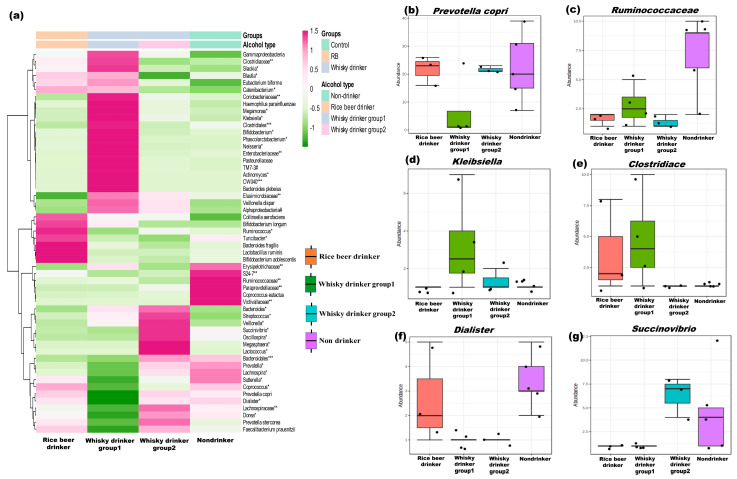
Altered microbial species detected in the study: (**a**) heatmap with clustering dendrograms depicting the group-wise altered bacterial species (Euclidean distance and Ward’s linkage were used for plot generation). The color intensity representing the median relative abundances, is based on the—z scores. Box and whisker plots depicting the relative abundance of (**b**) *Prevotella copri*, (**c**) Ruminococcaceae, (**d**) *Klebsiella* sp., (**e**) Clostrideaceae, (**f**) *Dialister*, (**g**) *Succinovibrio* sp. ** *p* values are listed in Table S4. (* Genus, ** Family,*** Order, # Phylum).

Firmicutes, Bacteroidetes, Actinobacteria, and Proteobacteria were the main bacterial phyla found in all of the groups (Figure S4a–d). In comparison with the non-drinkers, the Firmicutes/Bacteroides ratio was considerably higher in the whisky type 1 group (% abundance in whisky type 1: 48.851 vs. non-drinkers: 1.348; *p* = 0.01, Figure S3e). Figure 2a depicts the altered species observed in the study. The whisky type 1 group was found to have a higher *Bacteroides*/*Prevotella* ratio than the non-drinkers (% abundance in whisky type 1: 19.779 vs. non-drinkers: 0.019; *p* = 0.01, Figure S3f).

Except for the whisky type 1 drinkers, *Prevotella copri* was the most prevalent bacterium in all volunteers (% abundance in non-drinkers: 30.556; whisky type 2 drinkers: 34.887; rice beer drinkers: 34.715 vs. whisky type 1 drinkers: 9.849, Figure 2a,b). Additionally, the amount of Ruminococcaceae was substantially lower in both types of whisky drinkers (Figure 2c).

The whisky type 1 group contained a higher abundance of *Klebsiella* and Clostrideaceae (Figure 2d,e, respectively). On the other hand, non-drinkers and rice beer drinkers were dominated by *P. copri* and *Dialister* (Figure 2b,f).

The distinctive gut flora of whisky type 2 drinkers included *Acidaminococcus* sp. and *Enterococcus cecorum* (% abundance: 0.007 and 0.004, respectively). Additionally, more *Succinovibrio* (Figure 2g) but less *Bifidobacteria* were noticed in this group (% abundance in whisky type 2: 0.001 vs. non-drinkers: 0.348, *p* = 0.01, Table S3).

### 3.2. Effect of Alcoholic Beverages on Mouse Gut Microbiome (Validation Phase)

**Figure 3:**
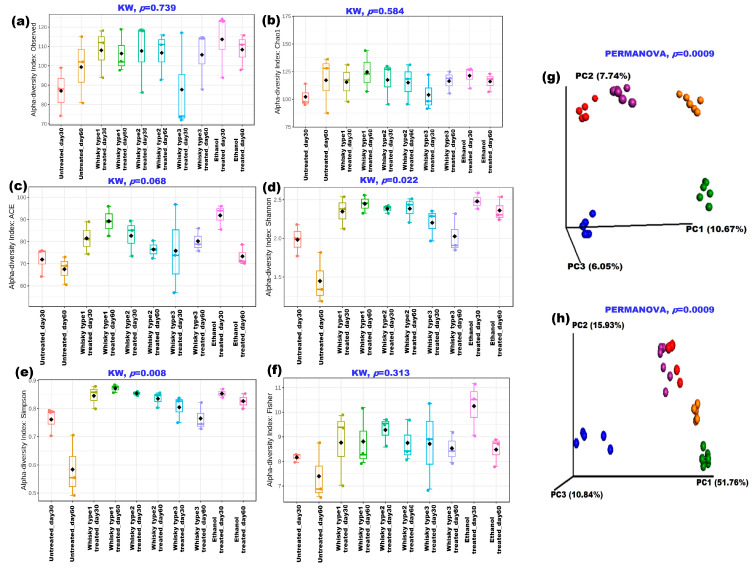
Effect of different whisky types in mouse gut bacterial profile: (**a**–**f**) α-diversity indices across the groups (Kruskal–Wallis test was performed to test the significant differences among the groups). ß-diversity of the groups in terms of unweighted (**g**) and weighted UniFrac (**h**) PCoA plots of fecal bacterial communities. {Color codes: **blue—**untreated/control, C; **green—**whisky type 1; **purple—**whisky type 2; **red—**whisky type 3; and **yellow—**ethanol treated}.

We next reproduced the parameter in a mouse model after examining the human group. Figure S5 presents DGGE-based observations of the mouse gut bacterial profile before and after the treatments (Figure S5a–e). The PCA plot based on the DGGE bands revealed the treatment-specific clustering of whisky types 1 and 2 after 30 days of treatment (Figure S5f). As depicted in Figure 3, the species richness did not differ significantly among the cohorts (Figure 3a–c); however, more species evenness in terms of Shannon, Sompson and Fisher diversity indices was observed in the treated groups (Figure 3d–f). Both weighted and unweighted UniFrac-based principal coordinate analyses (PCoA) revealed treatment-based clustering of the samples (*p* = 0.0009, Figure 3g,h).

Bacteroidetes, Firmicutes, and Proteobacteria were the three primary bacterial phyla that were found in all groups (Figure S6a). It is interesting to note that the OTUs mapped to the Porphyromonadaceae family in the RDP database correlate to the family S-24 in the Greengenes database. Fifty-one bacterial species were found to be altered across the treatment groups (Table S5).

In comparison with the untreated group, the Firmicutes/Bacteroidetes ratio was higher in the whisky type 3 cohort on the 60th day (Figure S6b,c, *p* = 0.03). The whisky type 1 group consistently had the highest *Bacteroides*/*Prevotella* ratio (30th day, type 1: 10.609 vs. C: 0.696, 60th day, type 1: 3.835 vs. C: 0.179; *p* = 0.01; Figure S6d).

**Figure 4:**
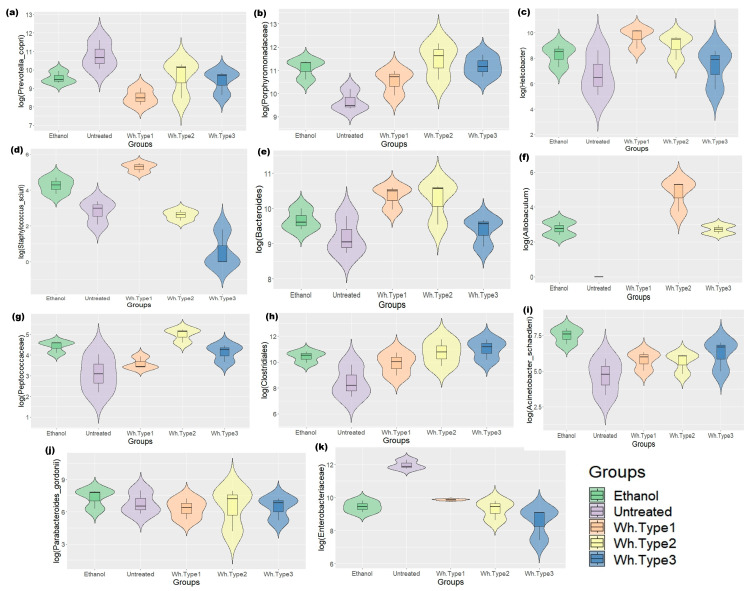
Box and whisker plots depicting the significant altered bacterial species in mouse study on day 60: (**a**) *P. copri* (*p* = 0.001), (**b**) Porphyromonadaceae (*p* = 0.007), (**c**) *Helicobacter* (*p* = 0.01), (**d**) *Staphylococcus sciuri* (*p* = 0.001), (**e**) *Bacteroides* (*p* = 0.003), (**f**) *Allobaculum* (*p* = 0.005), (**g**) Peptococcaceae (*p* = 0.02), (**h**) Clostridiales (*p* = 0.008), (**i**) *Acinetobacter schaedleri* (*p* = 0.003), (**j**) *Parabacteroides gordonii* (*p* = 0.06), and (**k**) Enterobacteriaceae (*p* = 0.001). {untreated—mouse group without any treatment, Wh.Type1—whisky type 1 treatment group, Wh.Type2—whisky type 2 treatment group, Wh.Type3—whisky type 3 treatment group, Ethanol—ethanol-treated group}.

Similar to that in the human groups, *P*. *copri* was found to be reduced in the treatment groups (Figure 4a, *p* = 0.001). Additionally, treatment-specific differences were observed in the mice microbiome. The whisky groups were dominated by Porphyromonadaceae in their gut compared with the untreated ones (Figure 4b). Specifically, similar to humans, the whisky type 1 group had significantly lower levels of *P. copri* (*p ≤* 0.001) and a higher abundance of *Helicobacter* sp. (% abundance on the 30th day: 10.356; on the 60th day: 10.520; *p ≤* 0.01, Figure 4c) compared to the other groups. In both study points, the group had considerably more *Staphylococcus sciuri* and *Bacteroides* (Figure 4d,e; *p* = 0.003). The whisky type 2 group was dominated by *Allobaculum* and *Peptococcaceae* (*p* = 0.007, 0.01; Figure 4f,g).

On the other hand, the whisky type 3 group exhibited a preponderance of unidentified bacterial clusters within the Clostridiales (Figure 4h, *p* = 0.008). The ethanol-treated group was dominated by *Acinetobacter schaedleri* and *Parabacteroides gordonii* (*p* = 0.003, 0.059, Figure 4i,j), while similar to the human study, the untreated mouse group was dominated by *P*. *copri* and Enterobacteriaceae (*p* = 0.001, Figure 4a,k).

### 3.3. Effect of Whisky Brands on Fecal and Plasma Metabolites in Animal Model

#### 3.3.1. Fecal Metabolites

**Figure 5:**
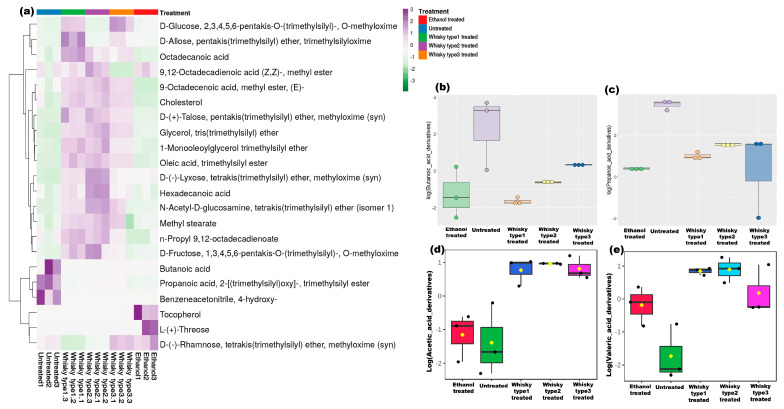
Fecal metabolome of mice on day 60: (**a**) heatmap depicting treatment-specific metabolite compositions. The top 10% metabolites detected in the study after 60 days of treatment were considered, and for heatmap generation, Euclidean distance, and Ward’s linkage were used. (**b**–**e**) Box and whisker plots showing significant alterations in SCFAs viz. butyric/butanoic acid, propanoic acid, acetic acid, and valeric acid in the groups.

Metabolites of known microbial origins or known to be influenced by microbes were focused in this study (Table S6). Short-chain fatty acids (SCFAs) were studied in the fecal samples collected on the 0th, 30th and 60th days of treatment. Significant clustering based on the treatments was observed on the 30th day and 60th day of treatments(Figure S7a–c). Figure 5a showed the most abundant metabolites (AUC ≥ 10%) across the groups. The SCFA levels were unaltered in the untreated group, while butanoic and propanoic acids were less abundant in the treatment groups after the 30th (Figure S8a–e) and 60th days of treatments (Figure 5b,c; *p* = 0.015; Table S6). Acetic and valeric acid levels were higher in the whisky-treated cohorts (Figure 5d,e; *p* ≤ 0.01; Table S6). A total of 293 metabolites were detected in the study (Table S8). Strikingly, we observed more metabolites on the 60th day followed by the 30th day, compared with the 0th day (Figure S9a–c); additionally, on day 0, we did not find any significantly altered metabolites among the groups (Figure S9d), while on day 30, we observed 67 metabolites that were significantly altered among the groups and, notably, 267 compounds were significantly altered in the groups on day 60 (Figure S9e,f). In this section, we mostly describe the metabolites observed on day 60. Apart from SCFAs, more sugars, sugar alcohol, and amino sugars were found in the treatment groups compared with the levels of these in the controls (*p* < 0.01, Figure S10a). In the whisky-treated cohorts, elevated levels of lipids were detected, while in the untreated group, fatty acids and carboxylic acids were more prominent (Figure S10). Specifically, 2-monopalmitoylglycerol, which is a known algal metabolite, trehalose, which is produced by *Saccharomyces cerevisiae*, were detected in the treated groups only (Figure S10c,l). Additionally, more metabolites of linoleic acid were found in the whisky treated cohorts, except for the whisky type 3 treated group (*p* ≤ 0.05). Inositol and myo-inositol have also been detected in the alcohol treated cohorts (Table S6). Metabolites such as hydroxy-butyric acid, hydroxy-phenylbutyric acid, methyl-keto-butyric acid, butanedioic acid, keto-isocarpic acid, and methyl-mandelic acid were only detected in the whisky-treated cohorts (Table S6). Derivatives of pentadecanoic acid were only detected in the type 2 treated group, whereas the metabolic marker pantothenic acid, which is contributed by intestinal bacteria, was only detected in the whisky type 1 treated group (Table S6). Notably, l-arabitol was detected only in the whisky type 1—whisky type 1 treated group (Table S5).

#### 3.3.2. Plasma Metabolites

**Figure 6:**
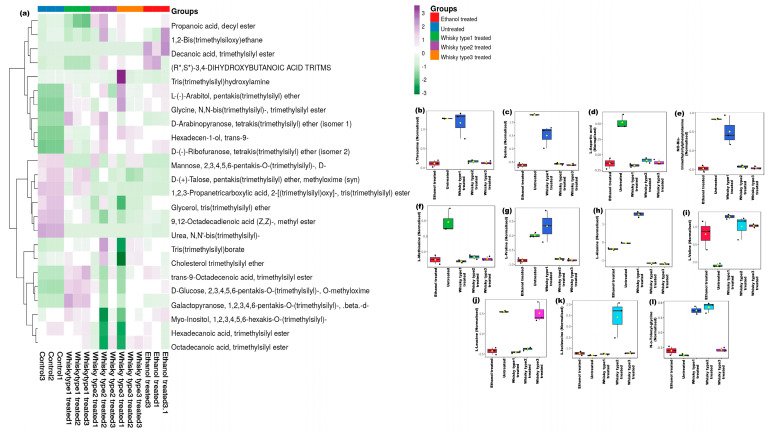
Plasma metabolome of mice on day 60 (MSTFA derivatized): (**a**) heatmap depicting treatment-specific metabolite compositions. Top 10% metabolites detected in the study after 60 days of treatment were considered, while for heatmap generation, Euclidean distance, and Ward’s linkage were used. Box and whisker plots showing significant alterations in (**b**) l-threonine (*p* = 0.02), (**c**) serine (*p* = 0.01), (**d**) l-aspartic acid (*p* = 0.02), (**e**) phenylalanine(*p* = 0.02), (**f**) l-methionine (*p* = 0.02), (**g**) l-proline (*p* = 0.02), (**h**) l-alanine (*p* = 0.02), (**i**) l-valine (*p* =0.02) (**j**) l-leucine (*p* = 0.02), (**k**) l-isoleucine (*p* = 0.02), (**l**) glycine (*p* = 0.01), concentrations in the groups.

**Figure 7:**
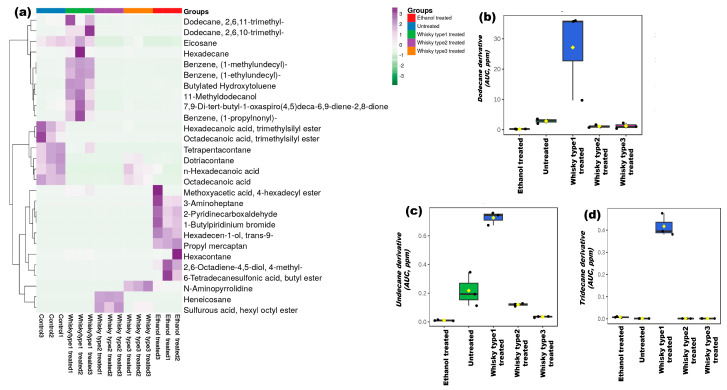
Plasma metabolome of mice on day 60 (BSTFA derivatized): (**a**) heatmap depicting treatment-specific metabolite compositions. Top 10% metabolites detected in the study after 60 days of treatment were considered, while, Euclidean distance, and Ward’s linkage were used in generating the heatmap. (**b**–**d**) Box and whisker plots depicting the altered stress markers noticed in the groups (KW, *p* = 0.05).

The GC-MS profiles based on both MSTFA and BSTFA derivatization showed distinct grouping on the basis of treatment (*p* ≤ 0.01) (Figure 6 and Figure 7). In total, 117 metabolites were significantly altered across the study groups, tabulated in Table S7 (Figure 6a and Figure 7a). We further considered amino acids and compounds altered in stress conditions for evaluating the differential effects of the beverages. More threonine, serine, aspartic acids—phenylalanine and methionine were found in the untreated group (Figure 6b–f). However, more proline, alanine and valine were found in whisky type 1 treated mice (Figure 6f–i).

The whisky type 2 group had more leucine and isoleucine, while glycine was found to be elevated in both the whisky type 1 and 2 groups (Figure 6j–l). The stress markers dodecane, undecane and tridecane derivatives were dominant in the whisky type 1 group (*p* ≤ 0.02) (Figure 7b–d and Table S7).

Apart from these metabolites, the levels of the alcoholic group of compounds were higher in the treatment groups (*p* ≤ 0.01, Table S7), and among the groups, the whisky type 2 group had higher levels of the alcohol group of compounds (% peak area- 4.407), followed by the type-3-treated (% peak area—4.163), ethanol-treated (% peak area—3.315), and whisky type 1 treated (% peak area—2.699) groups (*p* ≤ 0.05) (Table S7). More carboxylic and fatty acids were found in the untreated group (% peak area—1.183 and 17.711, respectively; *p* ≤ 0.02), while the type-3-treated group exhibited the lowest levels of these metabolites. The whisky-type-3-treated group had the lowest concentrations of carboxylic acid (% peak area—0.006%). Additionally, sugars were more abundant in the whisky type 1 group (% peak area—29.425), followed by the whisky type 2 cohort (% peak area—20.148; *p* ≤ 0.02, Table S7).

#### 3.3.3. Crosstalk between Host Microbiome and Metabolome

We then evaluated the crosstalk between the microbiome and metabolome of the host. For this purpose, we analyzed the correlation between significantly altered microbial species, plasma amino acids, and fecal SCFA concentrations on day 60. As shown in Figure S13, butanoic acid was found to a have negative correlation with all the significantly altered bacteria in the untreated, whisky type 1, and ethanol-treated groups (Figure S13a,b,e). However, in the whisky type 2 cohort, it positively correlated with *Burkholderia* (Figure S13c). In contrast, the whisky type 3 cohort had only positive correlations with butanoic acid and microbes such as *Bilophila*, *Streptococcus*, and *Bacteroides eggerthii*. (Figure S13d). There was no negative correlation observed between microbes and metabolites within the group. Furthermore, there was a strong negative correlation between propanoic acid derivatives and the microbes in the untreated group, while it was positively correlated with Enterobacteriaceae (whisky type 1), *Allobaculum* (whisky type 2), and *Prevotella* and *Citrobacter* (ethanol). Plasma amino acids, such as l-alanine, were found to be negatively correlated with *Prevotella*, *Lactobacillus ruminis* (whisky type 1), and Porphyromonadaceae, *P*. *copri* (ethanol). L-threonine was found to be negatively correlated with all significantly altered bacteria in the untreated and whisky type 1 treated groups (Figure S13a,b). With the exception of *Methylobacterium*, valine was observed to correlate adversely with all bacteria in the whisky type 2 group, including *Lactobacillus*, Porphyromonadaceae, *Prevotella copri*, *Bacteroides*, and *Helicobacter*. On the otherhand, *Burkholderia* and *Citrobacter* were discovered to have positive correlations with glycine. Leucine and valine, the two most prevalent plasma amino acids, were shown to have positive correlations with all of the significantly altered microorganisms in the whisky type 3 group (Figure S13d). Additionally, Alanine correlates negatively with *P*. *copri*, Porphyronadaceae, Enterobacteriaceae, etc. in the ethanol-treated group, while only unidentified species under the genus *Prevotella* sp. correlate positively with alanine in this group (Figure S13e). Valine, on the other hand, was found to have positive correlations with *Burkholderia* and *Pseudomonas*.

### 3.4. Effect of Whisky on Inflammatory Cytokines

Treatment of animals with different brands of whisky and ethanol caused a significant increase in the serum levels of IL1-β and TNF-α compared with the untreated animals (IL1 β (pg/mL)—untreated: 35.649, whisky type 1: 131.745, whisky type 2: 64.678, whisky type 3: 99.713, and ethanol-treated: 111.224—and TNF-α (pg/mL)—untreated: 0.097, whisky type 1: 0.461, whisky type 2: 0.241, whisky type 3: 0.336, and ethanol-treated: 0.426). Among all the treatment groups, the whisky type 1 group showed significantly higher levels of these inflammatory markers compared with the other groups (*p* ≤ 0.05, Figure S10).

### 3.5. Chemical Profiles of the Whisky Brands

**Figure 8:**
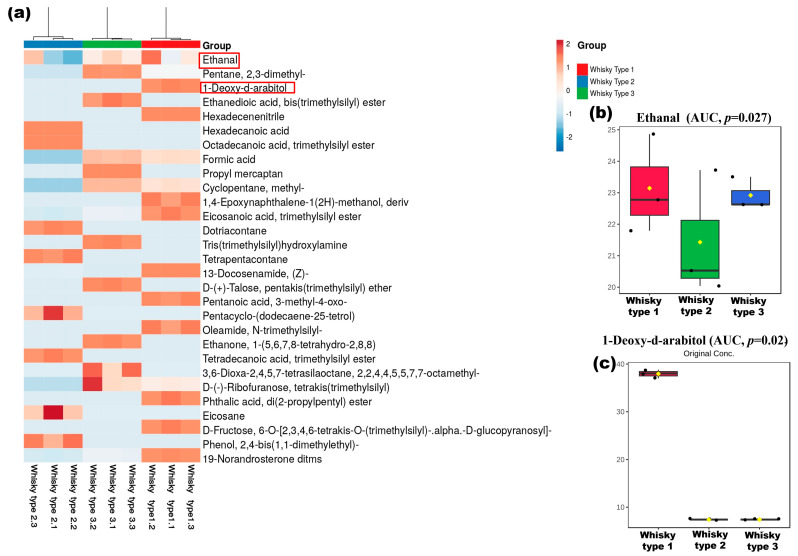
Chemical composition of the whisky samples studied. (**a**) Heatmaps were generated based on the chemical profiles of whisky brands. The top compounds with peak area ≥10% were considered (Euclidean distance and Ward’s linkage were used in generating the plot). Box and whisker plots present the original-concentrations of the most abundant compounds. (**b**) Ethanal/acetaldehyde (detected in all the samples) and (**c**) d-arabitol (which was predominant in whisky type 1) {Red box highlighted arabitol and ethanal concentration distribution across the studied samples}.

Upon observing the differential effect of alcoholic beverages on both the human and mouse gut microbiomes and metabolomes, we proceeded to analyze the chemical profiles of the whisky samples in parallel. We first evaluated the ethanol concentration in the three types of whisky and found similar ethanol compositions in them (Figure S12). Heatmaps generated based on the GC-MS data showed distinct clusters of the three brands (*p* ≤ 0.01, Figure 8). A total of 107 compounds were significantly different among the three whisky samples (Table S10). The alkane group of compounds such as derivatives of dodecane, dotriacontane, tetrapentacontane, cyclooctane, etc. were the major constituents that significantly differed in the three brands of whisky (*p* ≤ 0.01). 1-deoxy-d-arabitol, oleyl alcohol, heptafluorobutyrate, and pentanoic acid derivatives were present only in the whisky type 1 (M) (Table S8). The whisky type 2 (O) contained dotriacontane, tetrapentacontane, eicosane, and esters of propanoic acid and erucic acid only. Similarly, propyl mercaptan, derivatives of pentane, dodecane, octacosane, tetratetracontane, undecane, 1,2-benzenedicarboxylic acid, etc. were detected in the whisky type 3 (B) only. The profiles of sugar, alcohols, benzene compounds, ketones, and amines were significantly different in the whisky samples (*p* ≤ 0.01; Table S8). Sugar molecules were found in higher amounts in the whisky type 3 (B) samples (% peak area in B: 10.788 vs. M: 5.401 and O: 2.337; *p* ≤ 0.01), while more fatty acids were found in the whisky type 2 (O) (% peak area in O: 41.993 vs. M: 13.389 and B: 4.608; *p* ≤ 0.01). The whisky brand 3 (B), on the other hand, contained more amine groups of compounds, which were not detected in M (% peak area in B: 13.907 vs. O: 0.068; *p* ≤ 0.01; Table S8). Fructose was detected in the whisky brand 1 (M) only (5.401% peak area). Higher levels of alcohols such as derivatives of pentadecanol and hexadecanol were present in the whisky brand 2 samples, while decanol, 2-hexyl, and butane-1,3-diol were found only in the whisky brands 1 (M) and 2 (O), respectively (% peak area in M: 0.011 vs. O: 0.923). The fatty acids margaric and myristic acids were only present in the brand 1 samples (% peak area in O: 6.864 and 0.179), while more palmitic acid was detected in the whisky type 2 samples (32.059% peak area; *p* ≤ 0.03). Caprylic acid was only detected in the brand 3 samples (0.675% peak area). More stearic and oleic acids were found in both the brand 2 (O) and 3 (B) compared with the brand 1 (M) sample (*p* ≤ 0.02). Oxalic acid was the major carboxylic acid detected in the brand 3 (34.799%). More derivatives of phthalic acid were detected in the brand 1 (M) (% peak area in M: 6.267 vs. O: 0.872 and B: 0.025; *p* ≤ 0.04), and caproic acid was ubiquitously present in all three whisky brands (% peak area in M: 0.621, O: 0.204, and B: 0.505; Table S8).

Among these altered compounds, we then further focused on looking for the impact of the top 2 compounds, detected in whisky, specifically whisky type 1, viz. ethanal or acetaldehyde (present in all whisky), and arabitol (unique to whisky type 1) on the host gut microbiome (Figure 8b,c).

### 3.6. Effect of Arabitol and Ethanal on Gut Microbiome on the Mouse Gut Microbiome

**Figure 9:**
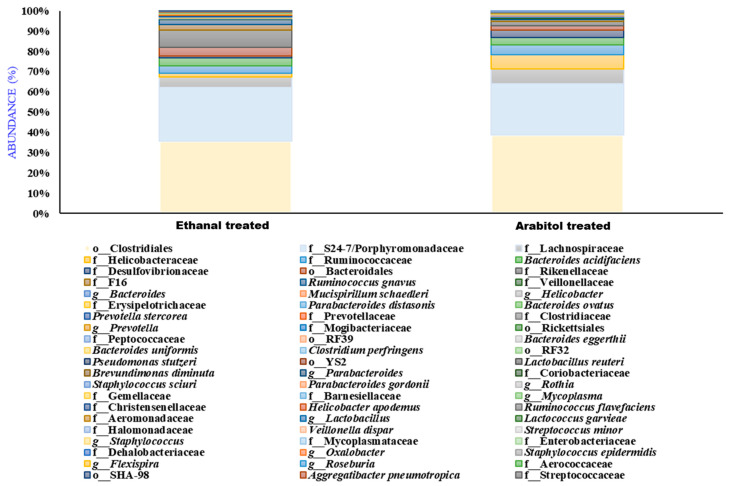
Bar diagram representing the bacterial species (%abundance ≥ 1%) detected in acetaldehyde/ethanal- and Ar/arabitol-treated groups.

We fed the 30% (*v*/*v*) arabitol and 20% (*v*/*v*) ethanal in sterile water to the mice via gavage for 21 days; then, evaluated its gut microbiome; and observed the elevated levels of Clostridiales and Porphyromonadaceae and reduced levels of *Prevotella copri* in both groups (Figure 9 and Table S9), which confirms our human and mice data of differential impact non-ethanolic compounds of the alcoholic beverages on gut microbiome.

## 4. Discussion

Alcoholic beverages contain diverse groups of non-alcoholic components. These non-alcoholic ingredients could differ according to the employed ingredients and preparation techniques. To assist the authorities in developing standards for the maintenance of the beverages’ quality, it is necessary to comprehend the impact of the non-alcoholic ingredients in these beverages on health. Previous research on wine’s non-alcoholic components showed that they had a favorable impact on gut microorganisms [22]. However, no research has focused on the non-ethanolic ingredients, along with the ethanolic concentrations in commercial alcoholic beverages, on the microbiome or health of the host. According to this study, the gut bacterial dysbiosis linked to whisky consumption is brand-specific in terms of its ingredients. The traditional 16S rDNA-based DGGE analysis of the gut microbiome distinguished the different drinker groups from one another, as well as from the non-drinker group. A fair conclusion could not be reached from these differentially shaped clusters because they were based only on 44 ribotypes (DGGE); hence, the samples were subjected to an NGS analysis. The three volunteer groups exhibited diverse gut bacterial profiles as a result of consistently consuming distinct whisky varieties over time, despite having the same amount of alcohol (42.8%, *v*/*v*). Therefore, it is extremely likely that alcoholic beverages’ non-ethanolic components, in addition to their ethanol levels, have a significant impact on the host microbiome. Variation between various varieties of wine or whisky is highly evident because these components come from diverse raw sources, production techniques, and additives. Manufacturing firms keep the chemical makeup and the specifics of the production procedures a secret. Therefore, the chemical composition of the alcoholic beverages used in this study will be helpful in explaining their differential effects on host microbiome.

Our microbiome analysis revealed that the drinkers of whisky group 1 had a different pattern of gut microbiome compared with whisky group 2. An increase in the F/B ratio in whisky group 1 may be an indication of the development of host inflammatory responses [23]. Additionally, one of the key observations in this study is, similar to a previous report, the significant reduction in *Ruminococcaceae* levels in the whisky drinkers (based on NGS data), which are mainly known as butyric acid producers [24]. Butyrate—one of the three major short-chain fatty acids (SCFA) formed in the colon, is the preferred energy source for colonocytes and has an activating effect on the apoptotic pathway, hence aiding in the prevention of intestinal diseases including colon cancer [25,26]. *Ruminococcaceae* has previously been shown to be linked with four enzymes involved in the thiamine biosynthesis pathway [27], and a deficit may lead to the development of an alcoholic brain disease, Wernicke–Korsakoff syndrome [28]. The family was also correlated with TNF-mediated anti-tumor responses [29]. There are reports of six enzymes in the haem biosynthesis pathway that are over-represented in *Ruminococcaceae* (enterotype 3), and therefore, a decrease in their abundance may affect the health of individuals [27,29].

Increased levels of Clostridiales and *Klebsiella* in whisky type 3 mice may indicate an increased risk of colorectal cancer, chronic atrophic gastric acid, and inflammatory colons [30,31,32]. Moreover, decreased abundance of *Prevotella* in the whisky type 1 group could lead to the production of less propanoic acid, which is essential in maintaining our health [33]. The health-promoting commensals, *Bifidobacteria*, which was depleted in the whisky type 2 group, has been linked to vitamin biosynthesis, digestion and absorption, a reduction in pathogen colonization, and stimulation of immune responses [34]. Similar to the human study, in the mouse study, whisky-brand-specific gut bacterial dysbiosis was observed. *Prevotella* was shown to be depleted in the whisky groups in both humans and mice, and among the whisky groups, the whisky type 1 group had considerably fewer *Prevotella* in their guts (*p* = 0.01). Whisky type 1 had a few unique compounds such as arabitol and fructose. Fructose and other sugar substitutes, such as sugar alcohols (such as xylitol), have been shown to have a major impact on the gut bacterial ecology [35]. *Prevotella* was found to be elevated in sugar-alcohol- and xylitol-rich diets [35,36]. However, in this study, the decreased abundance of *Prevotella* in the whisky type 1 group could be the effect of other compounds present in the whisky. Excessive fructose consumption has also been found to induce hepatic steatosis in a mouse study [37]. *Prevotella* had also been reported to be depleted in the patients of steatosis [38]. The family *Porphyromonadaceae* was found to be elevated in the alcoholic groups though its abundances varied across the treated cohorts. The role of *Porphyromonadaceae* in human health is still not clear, yet some seminal studies claimed its prevalence in colon carcinogenesis [39]. It has also been reported as an abundant flora in cirrhotic patients and alcoholic groups [40,41]. However, the abundance of *Porphyromonadaceae* in the whisky-treated groups might be due to its ability to ferment the ingredients present in the whiskies [42]. *Porphyromonadaceae* could also be linked to the elevated expression of TNF-α in the groups positively, as previously described [43]. The *Clostridial* clusters have been reported to be associated with both good and bad effects on the host [44]. However, due to our limited knowledge on the mouse gut bacterial profile, we are unable to describe the role of an abundant cluster of *Clostridiales* in the whisky-treated groups in this study. Additionally, the abundance of *Bacteroides* in the whisky type 1 cohort might be due to the presence of fructose in the whisky 1 group [45]. Elevated IL-1β expression has also been found in the whisky type 1 treated cohorts, which might be associated with the abundance of *Bacteroides*, as previous reported [45,46]. *Helicobacter* was found to be elevated significantly in the group, which could be due to its ability to ferment arabitol, which is present in whisky type 1 [47,48], and it is further correlated with an increased level of alanine in this group [49]. *Helicobacter* is a known pathogen and has also been linked with the elevation of IL1-β, tumor necrosis factor (TNF)-α expression, and carcinoma [50,51,52,53]. Additionally, the dominance of *Bacteroides* sp. can be linked to the detection of higher lipids in the fecal metabolite profiles of the respective group [54].

Concomitantly, in the profiles of both the fecal and plasma metabolites, more alcohol compounds were noticed in the treated groups than in the untreated ones. The stress markers dodecane, undecane, tetradecane, and tridecane derivatives were detected in the plasma of the whisky type 1 treated group which might be linked to the dominance of *Helicobacter* in that group [55]. The abundant *Clostridial* cluster may be linked to the abundance of glycine in the treated groups [56,57]. The lipid profiles were high in the whisky type 1 group, which might be associated with the high *Firmicutes*/*Bacteroidetes* ratio [58]. Although the effects of both whisky and pure ethanol were evaluated in the host gut, the differential responses to different whisky brands indicated the influence of the non-ethanolic components on both the gut bacteria, and the fecal and blood metabolomic profiles. Whisky type 1 had a few unique compounds such as d-arabitol, which were correlated with the abundance of *Helicobacter* sp., and l-arabitol was detected in the fecal metabolite profile of the treated group. Noticeably, whisky brand 1 had more sugars in its chemical profiles, and concomitantly, higher concentrations of sugars were noticed in the plasma metabolome of the treated animals. Our microbiome and metabolite data suggest that the reduced amount of butyrate producers, along with a reduced amount of butyrate/butanoic acid in the alcohol cohorts, could potentially lead to a disruption in the gut barrier integrity since butyrate is necessary for epithelial cell regeneration. Additionally, the elevated levels of stress markers and altered plasma amino acids might also exert a potentially harmful impact on the gut barrier integrity. However, we need to carry out more mechanistic evaluations including a larger longitudinal study with human subjects to obtain a concrete understanding of this aspect. Moreover, we also assessed the interactions of our microbe–metabolites profiles but did not observe any butyrate producers correlating positively with butanoic acid in the untreated groups, which needs further *in-depth* evaluations to understand the microbial crosstalk with the metabolites.

## 5. Conclusions

This study has revealed for the first time that non-alcoholic whisky components may considerably affect drinkers’ gut bacteriome/microbiome. This finding may apply to other alcoholic beverages as well, although more research is needed to help regulatory authorities monitor the quality of alcoholic beverages. Since different whisky brands contain different non-alcoholic ingredients, it is crucial to identify the ones that have a negative impact on host health and gut bacterial dysbiosis.

Our research has several restrictions. To determine the potential negative impacts of harmful bacteria on the host, we did not examine the amounts of LPS (lipopolysaccharide), tight junctional protein expression, or intestinal permeability. However, we instead took a thorough untargeted metabolomics approach to associate the metabolites, which are the by-products of all metabolite pathways in an association with host gut bacterial dysbiosis and its relationship to host health.

## Data Availability

The raw data were submitted to SRA under project ID PRJNA931847.

## Supplementary Materials

The following supporting information can be downloaded at https://www.mdpi.com/article/10.3390/microorganisms11061501/s1, Figure S1: Flowchart of the parameters considered for this study. Figure S2: DGGE profiles of the groups. (a) First trial experiment, (b) Second experiment with F5 progeny. Figure S3: Denaturing gradient gel electrophoretic (DGGE) profile of the V6–V8 region of the 16S rDNA of fecal DNA samples of the volunteers (codes: ‘Ref’, reference lane). Marked bands were sequenced and identified (tabulated in Table S2). Figure S4: Gut bacterial profiles of the participants. (a–d) Pie charts displaying phylum-level microbial abundances in the groups, (e,f) Box and whisker plots depicting the Firmicutes/Bacteroidetes ratio and *Bacteroides*/*Prevotella* ratio observed in the groups. Figure S5: Gut bacterial profile of different groups. (a–e) DGGE profiles of the mice on the initial day (i.e., day 0) and after 30 days of treatment. (f) Principal component analysis plot based on the DGGE profiles. Figure S6: Gut bacterial profile of different groups. (a) Heatmap depicting the microbial phyla detected in mice on day 30 and day 60, (b,c) Firmicutes/Bacteroidetes (F/B) ratio observed in the groups on day 30 and day 60, (d) *Bacteroides*/*Prevotella* ratio observed in the groups. Figure S7: PLSDA plot generated based on fecal metabolite concentrations on (a) day 0, (b) day 30, and (c) day 60 of the treatments. Figure S8: Short-chain fatty acid concentrations on the initial day, i.e., day 0—(a) Butanoic/butyric acid, (b) propanoic acid, and (c) acetic acid (*p* = 0.1, KW)—and on day 30, i.e., after 30 days of treatment—(d) butanoic acid (KW, *p* = 0.015) and (e) propanoic acid (*p* = 0.01, KW). Figure S9: Heatmap showing fecal metabolome of mice on (a) day 0, i.e., without any treatments; (b) day 30th, i.e., after 30 days of treatment; and (c) day 60. Dot plot depicting altered metabolites selected by Kruskal–Wallis tests with *p*-value threshold 0.05 (d) on day 0, (e) on day 30, and (f) on day 60. Figure S10: Significantly altered fecal metabolites (classes of compounds) on day 60. Figure S11: Effect of alcoholic beverages on circulatory cytokine levels. (a) IL1-β profiles and (b) TNF-α profiles of the mice. All the results were expressed in mean ± SD (n = 3) at *p* ≤ 0.05. C—control, M—whisky type 1; O—whisky type 2, B—whisky type 3, and E—ethanol (42.8%)-treated groups. Figure S12: Ethanol percentage of the whisky-type samples studied in this work. Figure S13: Network plot based on the correlation observed between microbiome and metabolome in (a) untreated, (b) whisky type 1, (c) whisky type 2, (d) whisky type 3, and (e) ethanol-treated groups (**green**—positive interactions, **red**—negative interactions, r = −1.0 to 1.0; *p* = 0.01). Table S1: Details about the volunteers. Table S2: Partial sequences of 16S ribosomal RNA gene of uncultured bacteria submitted in NCBI. Table S3: Effect of alcoholic beverages on gut microbial profiles of human cohorts (1% abundance). Table S4: Altered microbial species detected in the human study (LeFSE). Table S5: Significantly altered microbial species in mice. Table S6: Effect of alcoholic beverages on mice fecal metabolites. Table S7: Effect of alcoholic beverages on mice blood metabolites. Table S8: Chemical profiles of the whisky samples studied in the work. Table S9: Effect of arabitol and ethanal on gut microbiome on the mice gut microbiome.
