# Supplementary material for: Multi-Omics Analysis Demonstrates the Critical Role of Non-Ethanolic Components of Alcoholic Beverages in the Host Microbiome and Metabolome: A Human- and Animal-Based Study
Source: Microorganisms. 2023 Jun 5;11(6):1501. doi: 10.3390/microorganisms11061501 (PMC10301968; doi:10.3390/microorganisms11061501)
Supplement: Supplementary file 1 [file microorganisms-11-01501-s001.zip › microorganisms-2345143-supplementary.docx]

Supplementary Figures


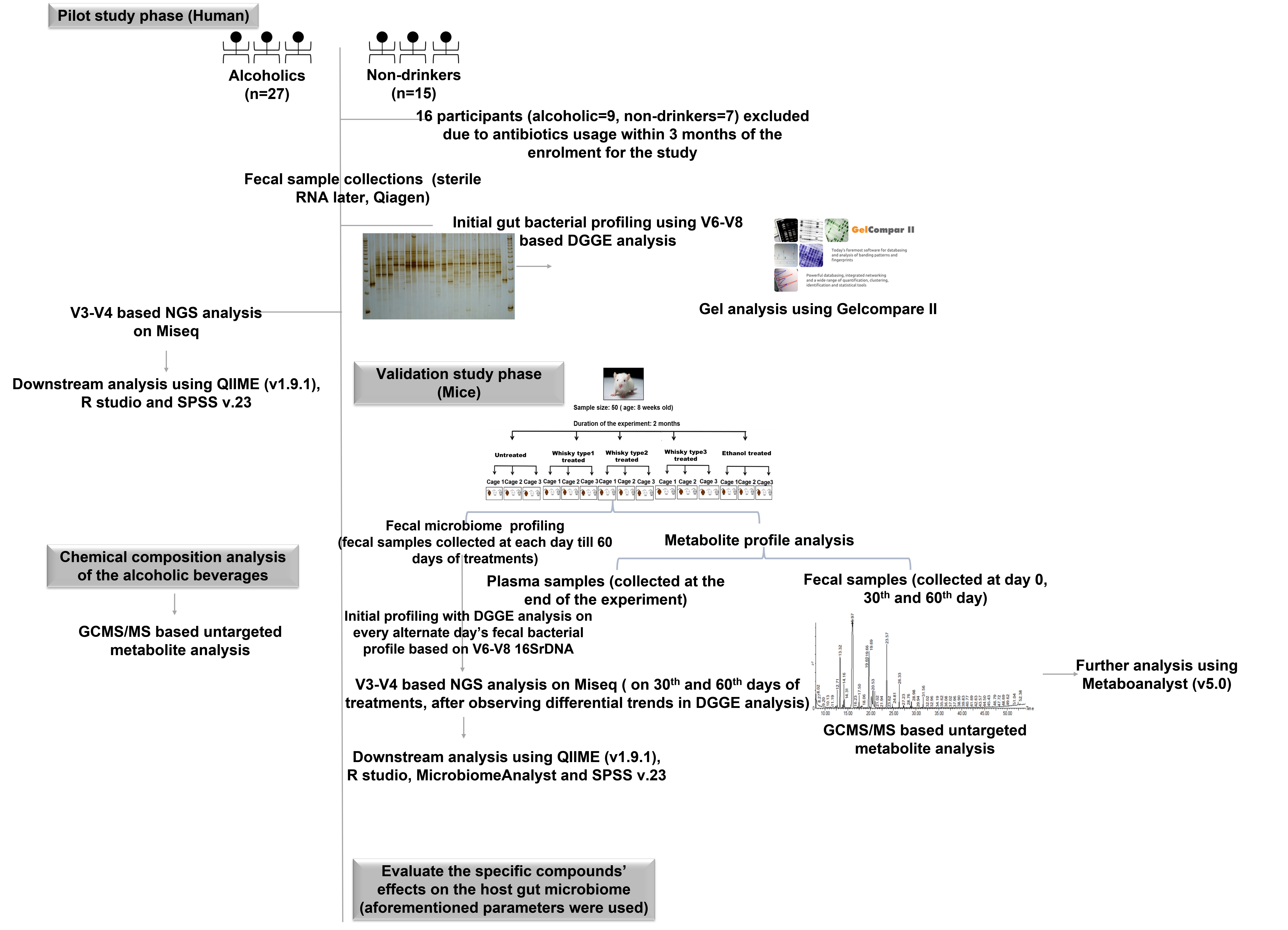


**Figure S1.** Flowchart of the parameters considered for the study.


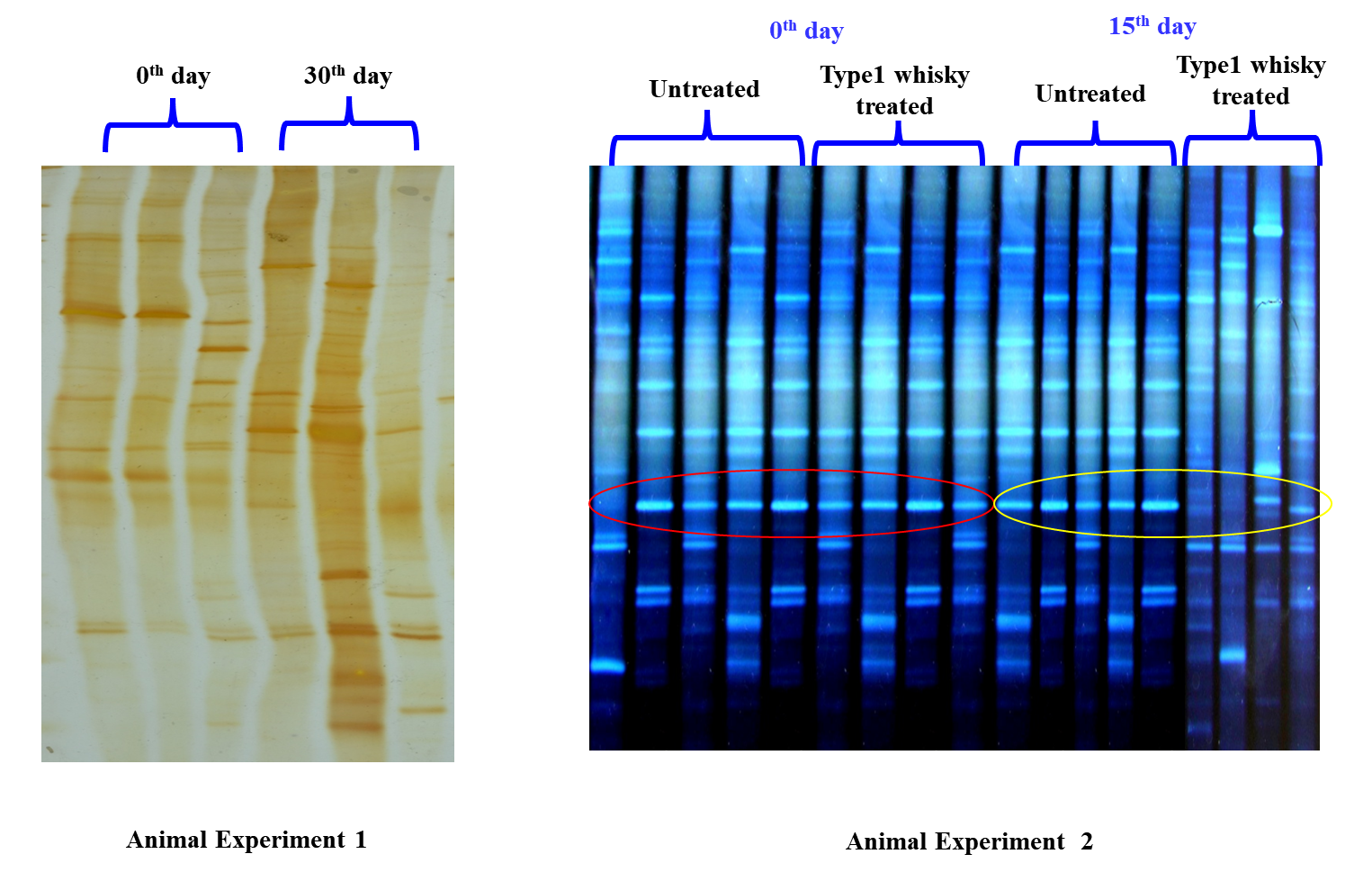


**Figure S2.** DGGE Profiles of the groups (a) First trial experiment, (b) Second experiments with F5 progeny.


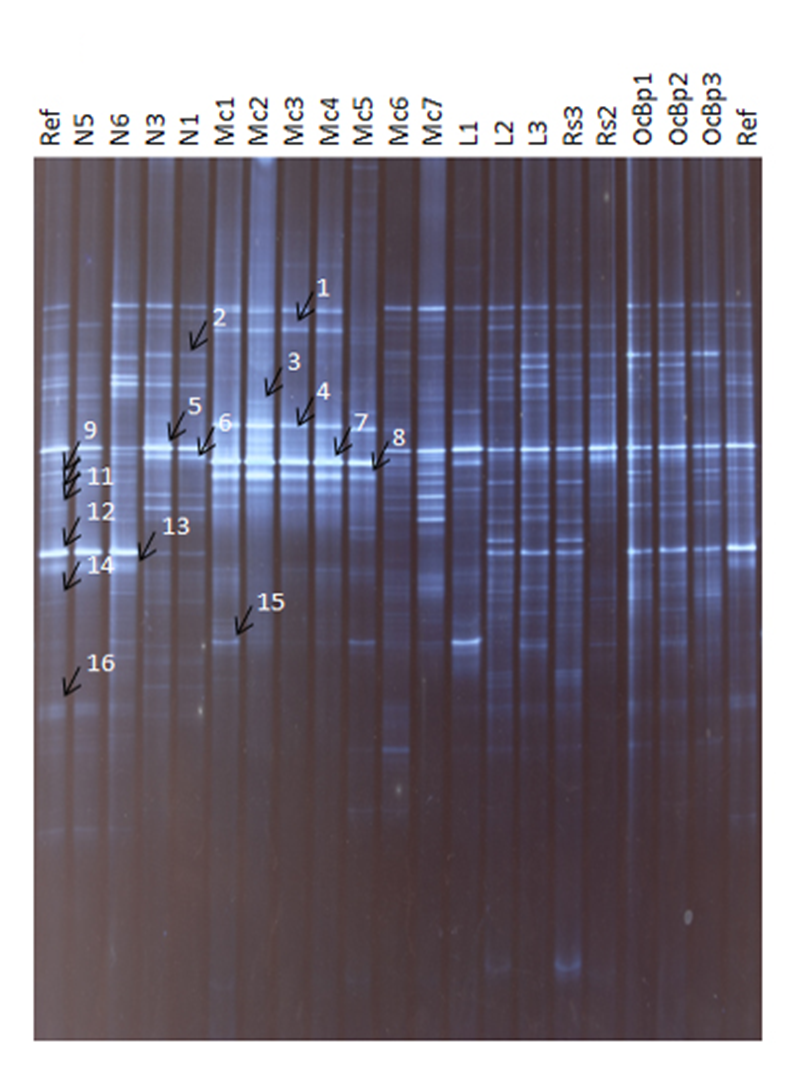


**Figure S3.** Denaturing gradient gel electrophoretic (DGGE) profile of the V6-V8 region of the 16S rDNA of fecal DNA samples of the volunteers (Codes: ‘Ref’, reference lane). Marked bands were sequenced and identified (tabulated in the Table S2).


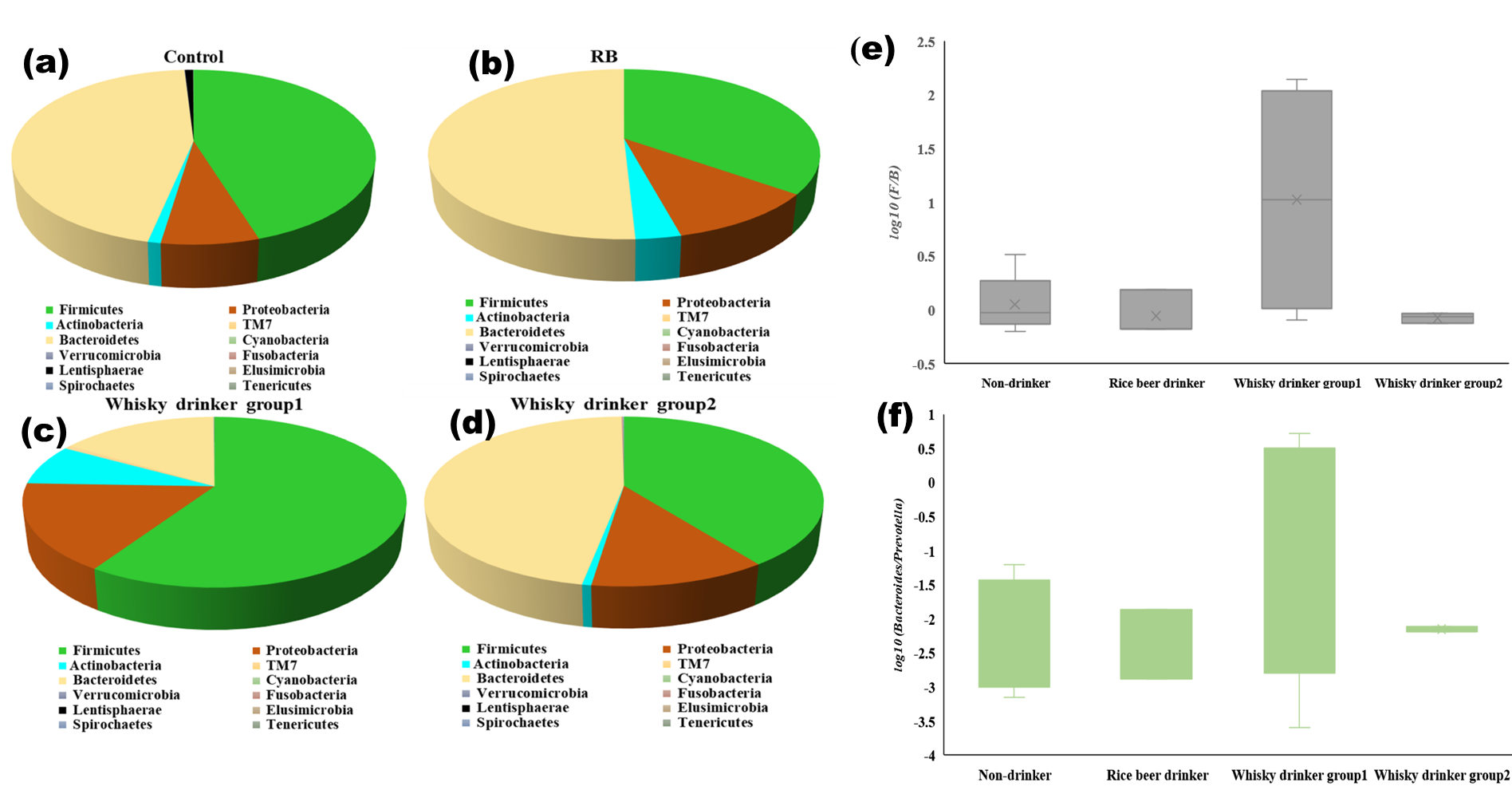


**Figure S4.** Gut bacterial profiles of the participants. (a-d) Pie charts displaying phylum-level microbial abundances in the groups, (e-f) Box and Whisker plots depicting the Firmicutes: Bacteroidetes ratio and *Bacteroides*:*Prevotella* ratio observed in the groups.


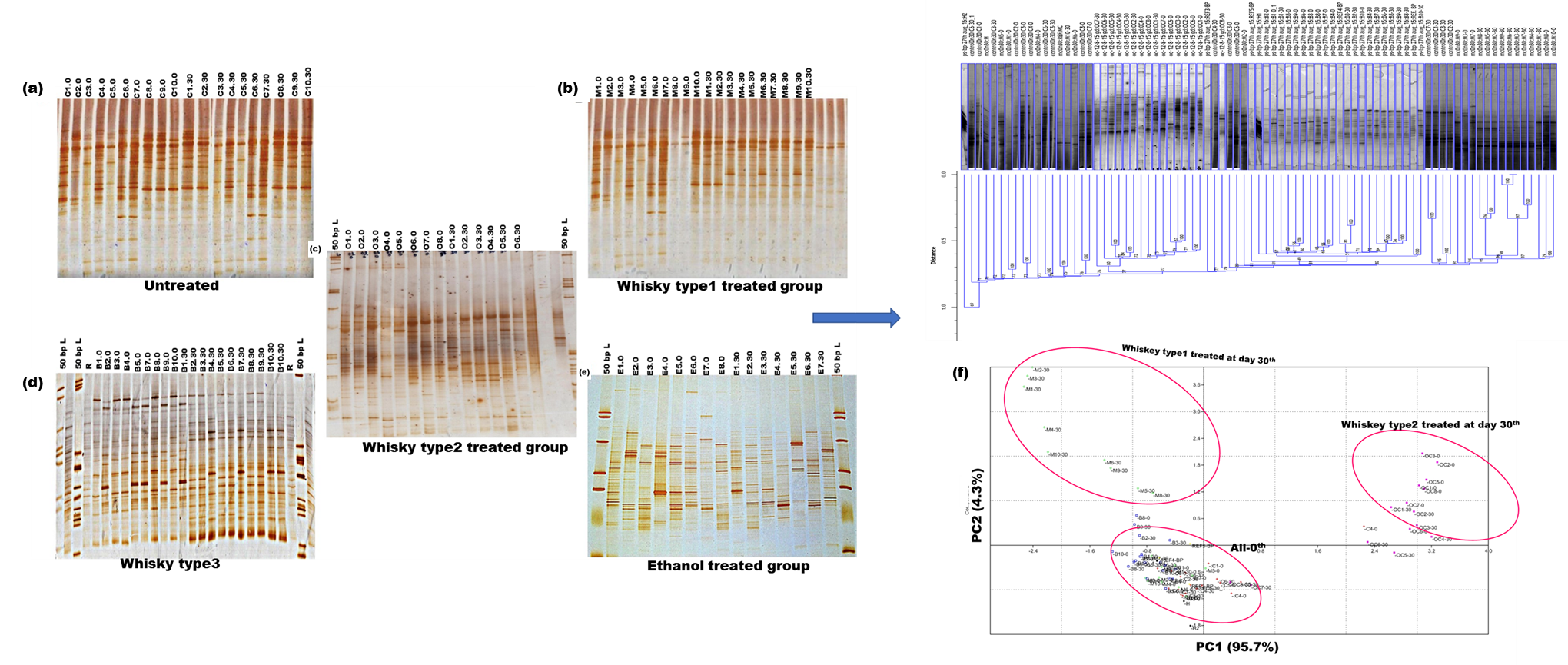


**Figure S5.** Gut bacterial profile of different groups (a-e) DGGE profiles of the mice from the initial day (i.e. day 0) and after 30th days of treatments (f) Principal Component Analysis plot based on the DGGE profiles.


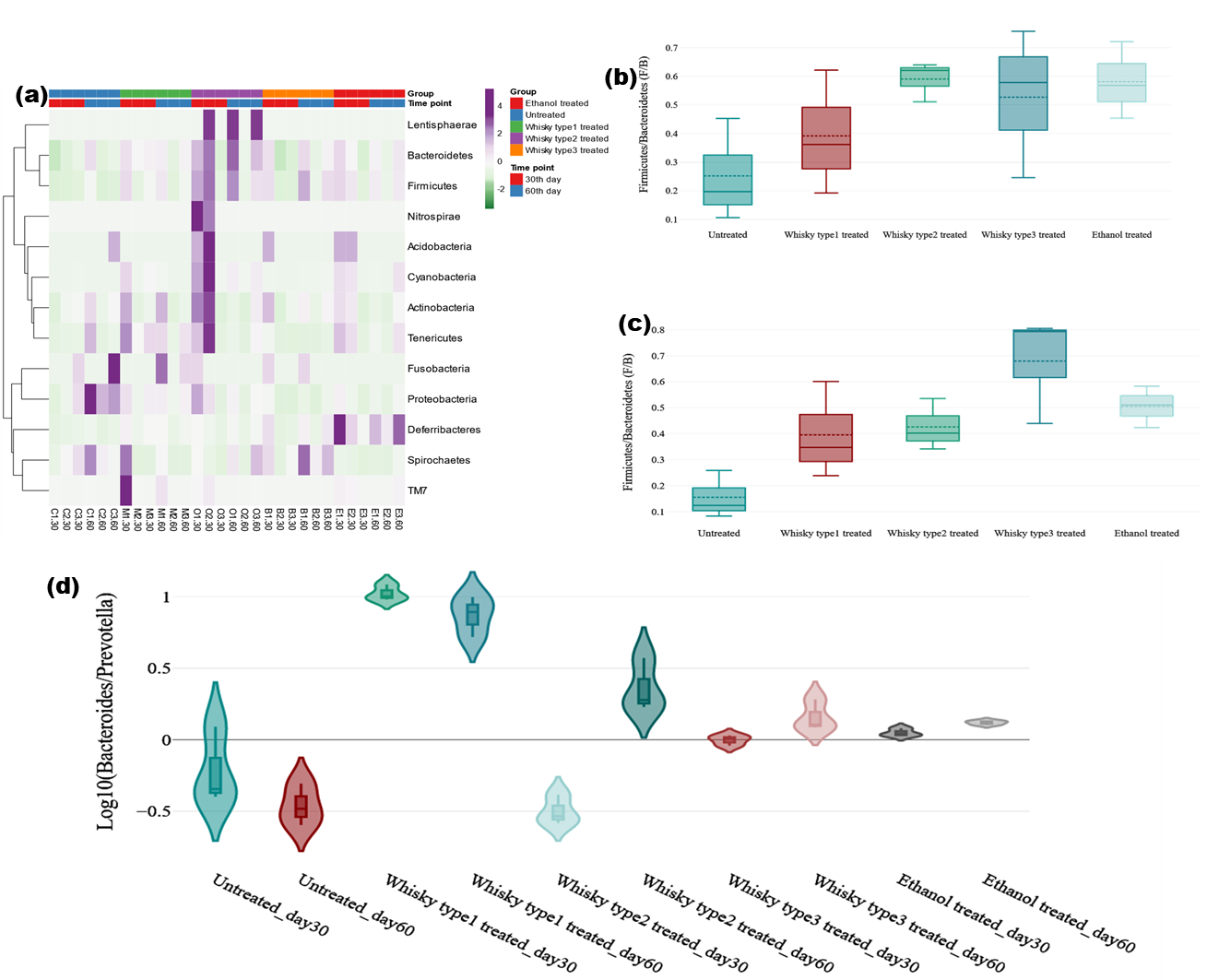


**Figure S6.** Gut bacterial profile of different groups (a) Heatmap depicting the microbial phyla detected in mice at day30 and day60, (b-c) Firmicutes/Bacteroidetes (F/B) ratio observed in the groups at day 30 and day 60, (d) *Bacteroides*/*Prevotella* ratio was observed in the groups.


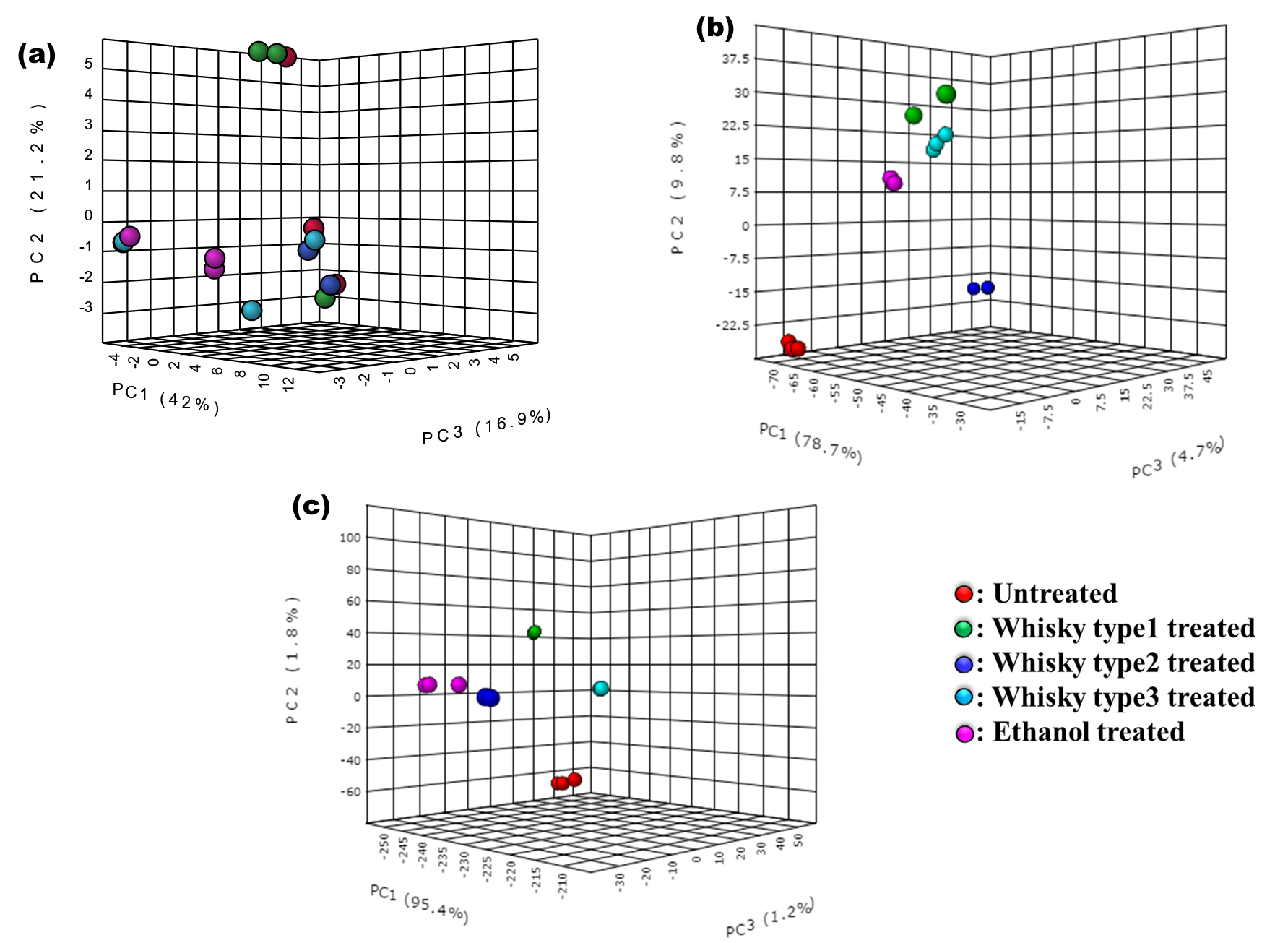


**Figure S7.** PLSDA plot generated based on faecal metabolite concentrations of (a) Day 0, (b) Day 30 and (c) Day 60th of the treatments.


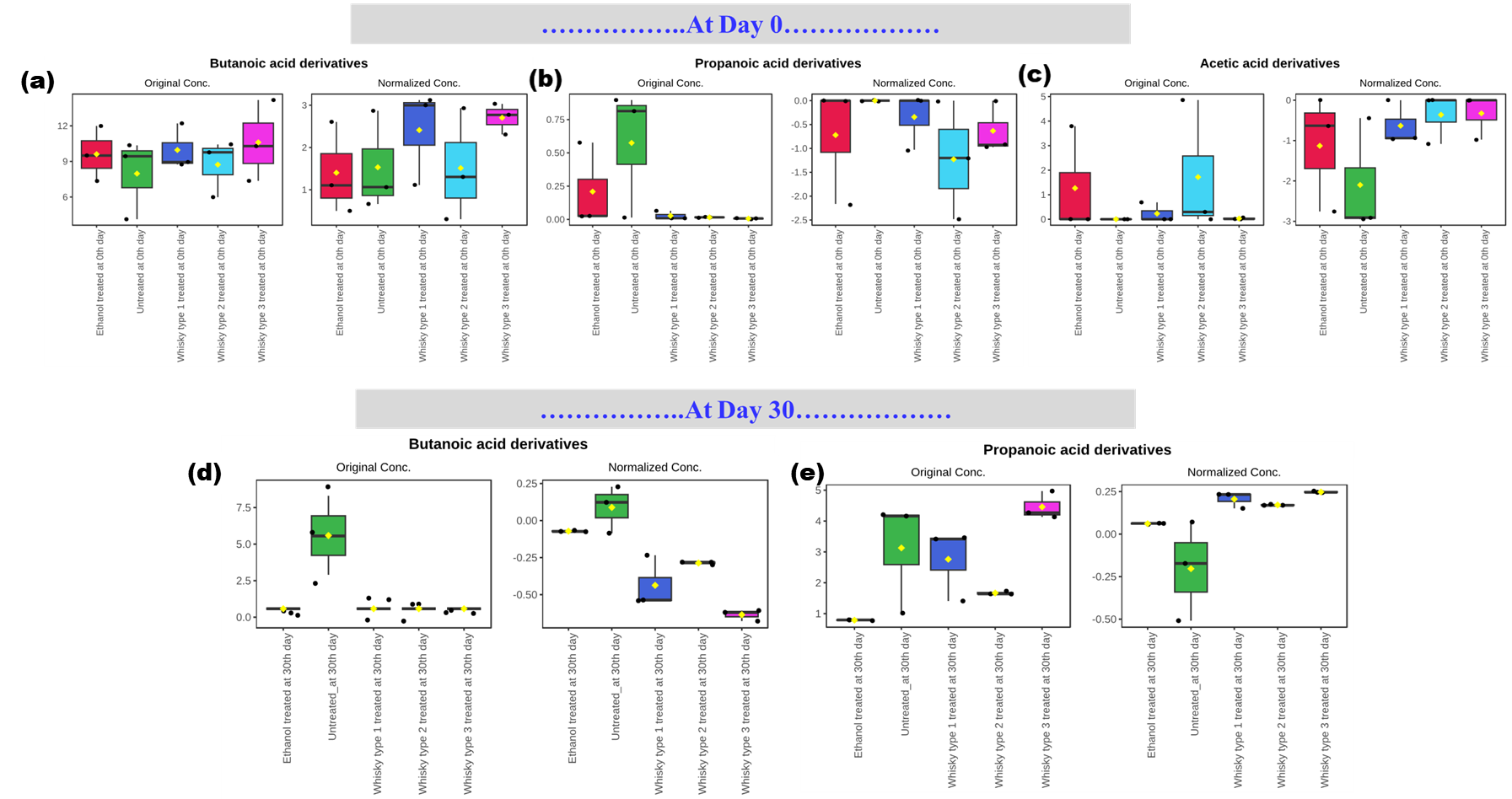


**Figure S8.** Short Chain Fatty Acids concentration in the initial day i.e. day 0 (a) Butanoic/Butyric acid, (b) Propanoic acid, (c) Acetic acid (*p*=0.1,KW) and Day 30 i.e. after 30days of treatments (d) Butanoic acid (KW,*p*=0.015), (e) Propanoic acid (*p*=0.01,KW).


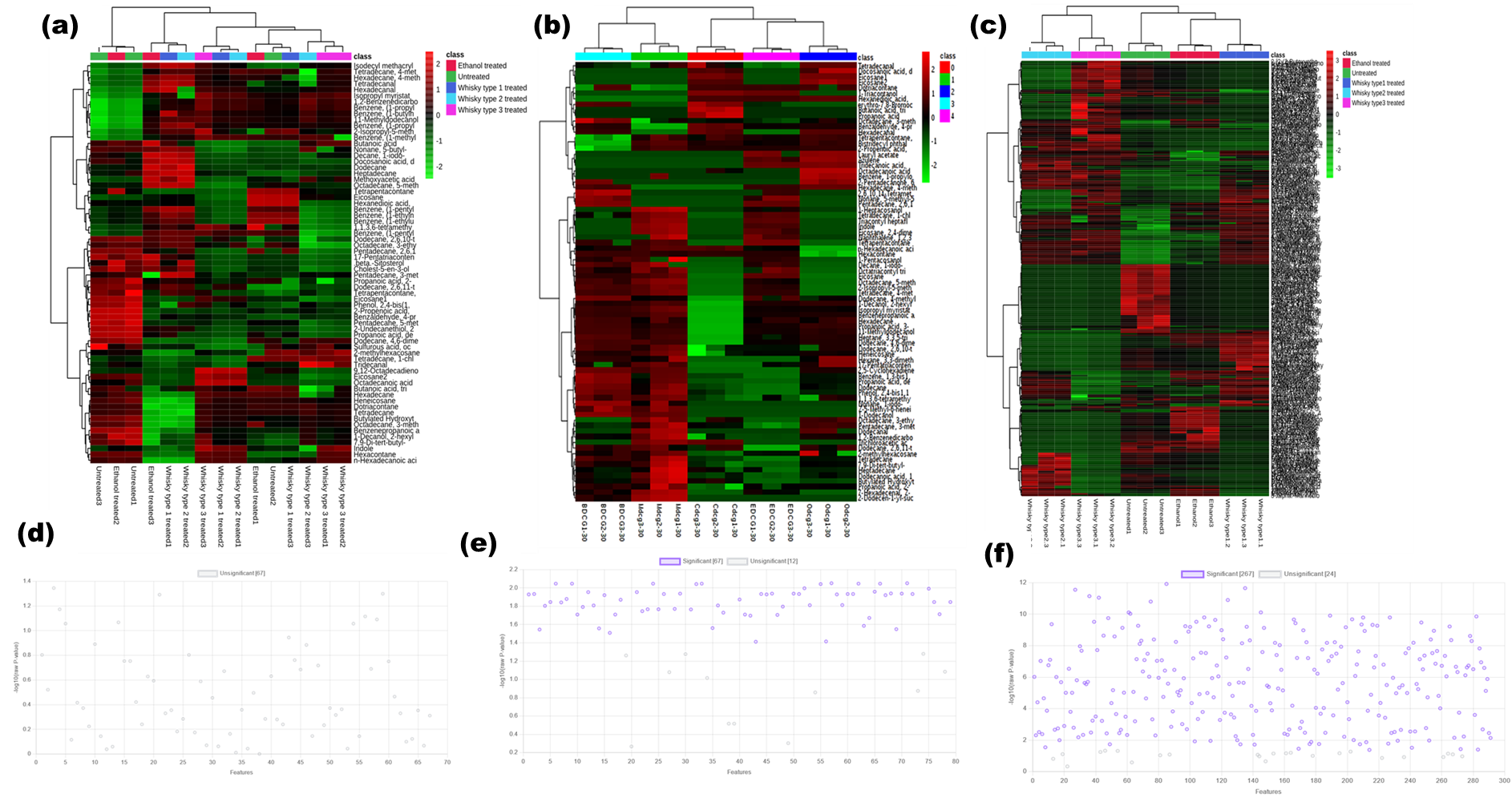


**Figure S9.** Heatmap showing fecal metabolome of Mice at (a) day0 i.e. without any treatments, (b)day 30th i.e. after 30days of treatmetns and (c) day 60. Dot plot depicting altered metabolites selected by Kruskal-Wallis tests with *p* value threshold 0.05 (d) at day 0, (e) at day30th and (f) at day 60^th^.


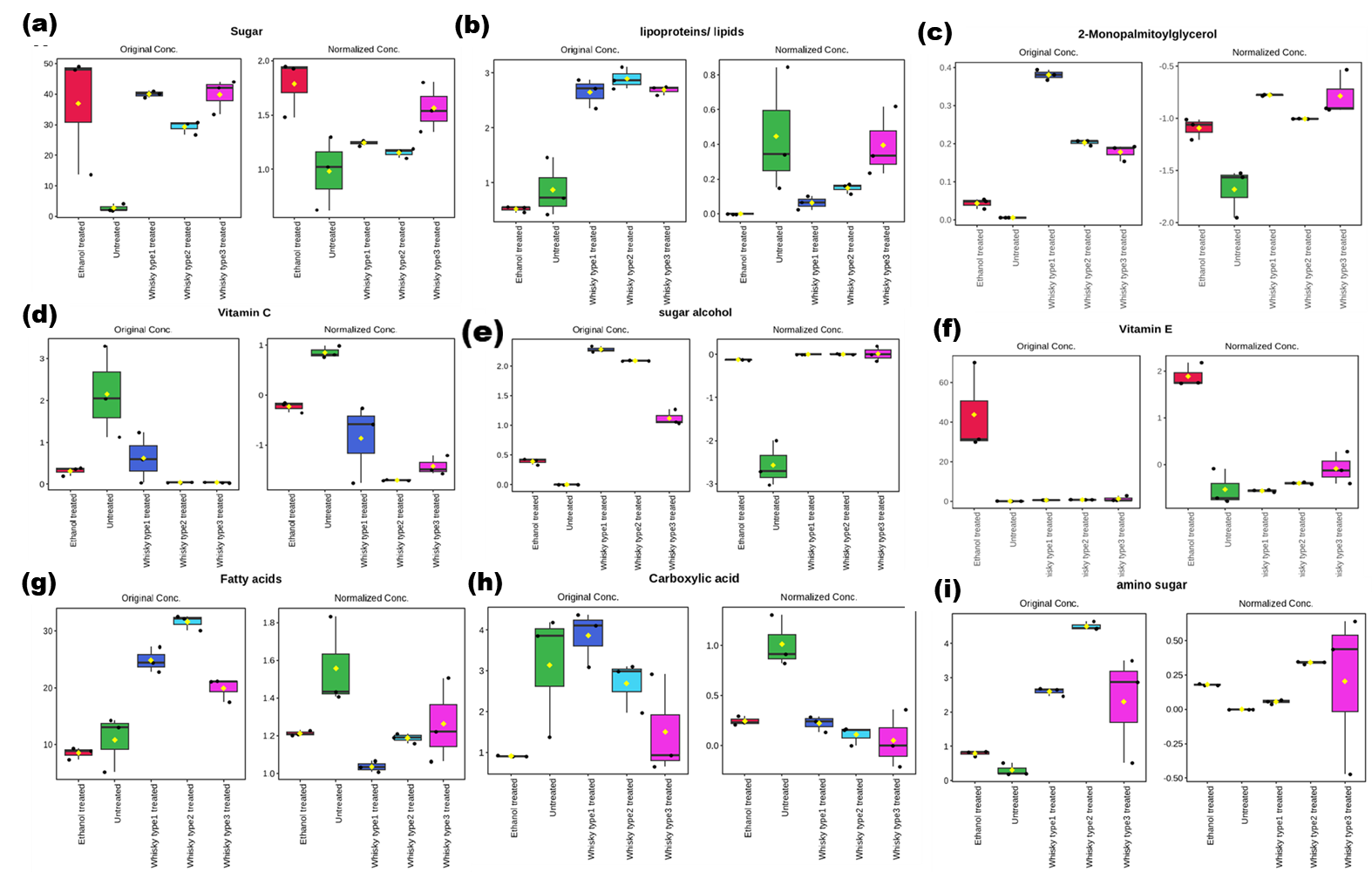


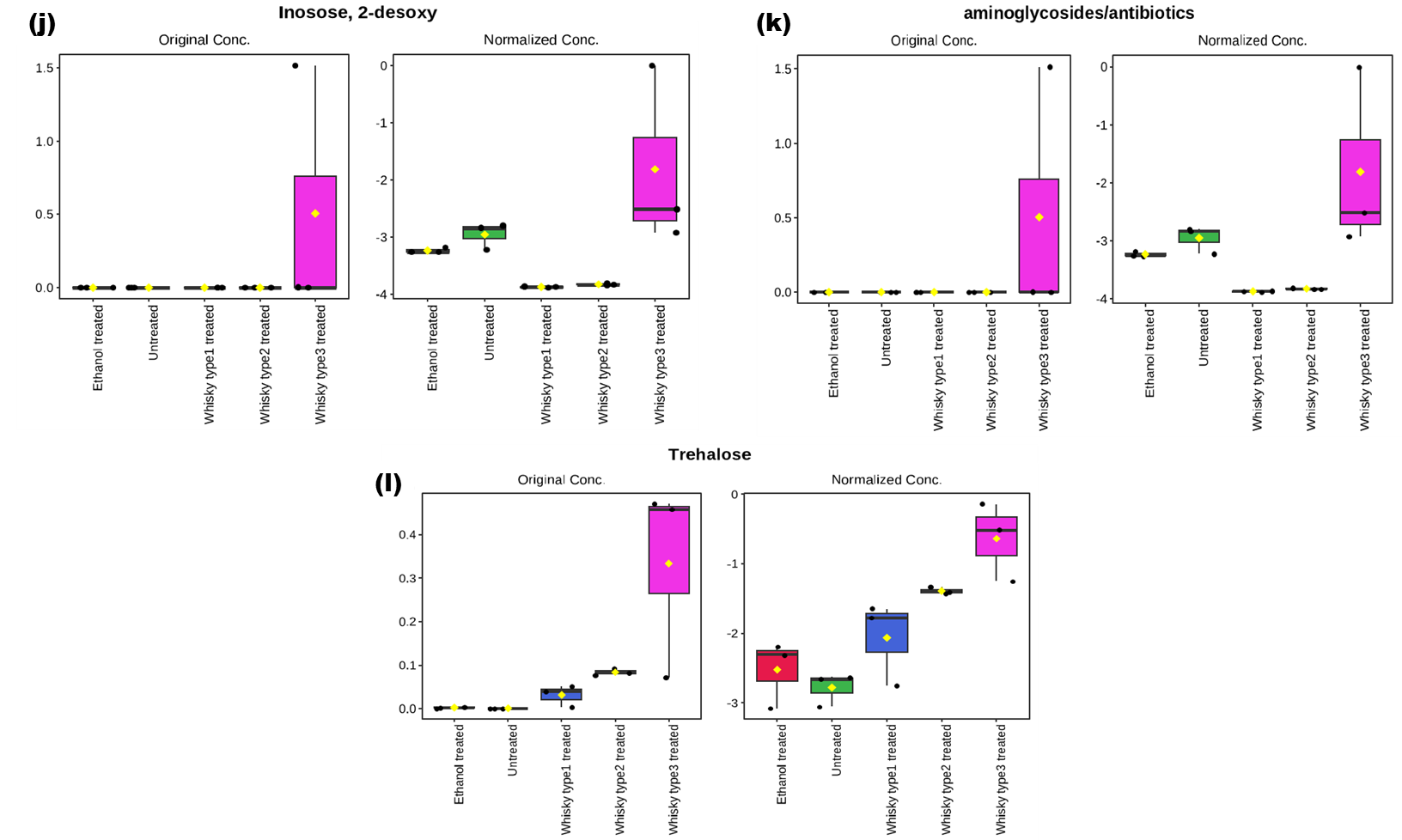


**Figure S10.** Significantly altered fecal metabolites (classes of compounds) at day 60^th^.


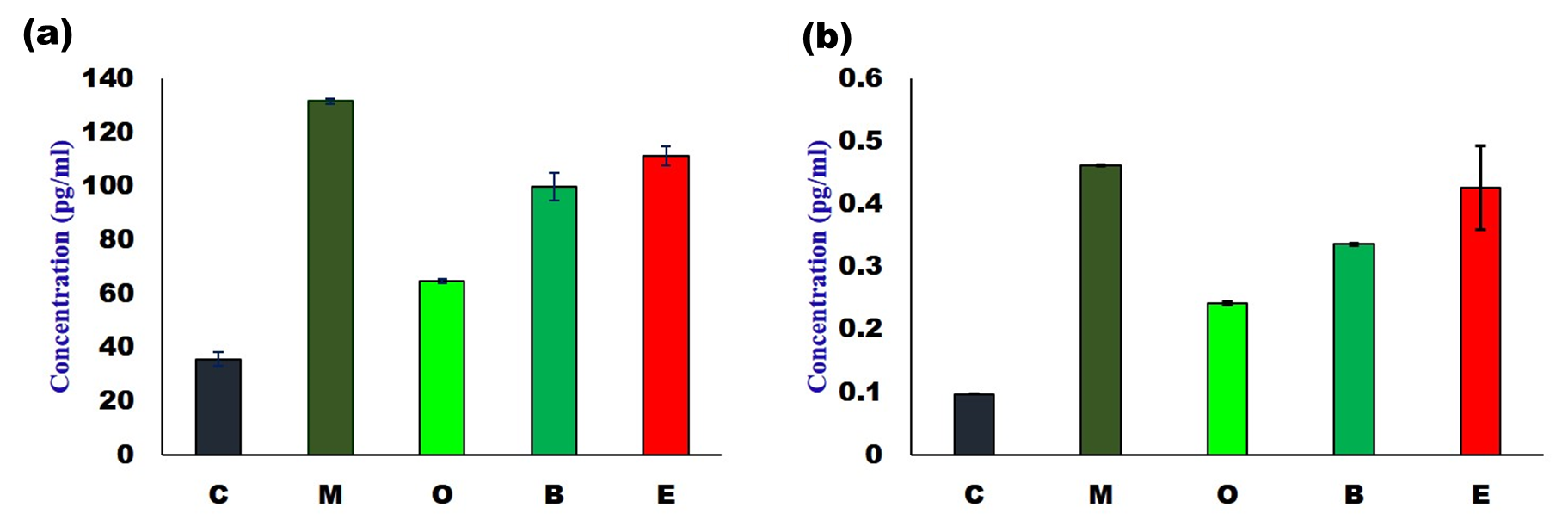


**Figure S11.** Effect of alcoholic beverages on circulatory cytokine levels. (**A**) IL1-β profiles and (**B**) TNF-α profiles of the mice. All the results were expressed in mean ± SD (n = 3) at *p*≤0.05. C - Control, M- Whisky type 1; O- Whisky type 2, B- Whisky type 3 and E- Ethanol (42.8%) treated groups.


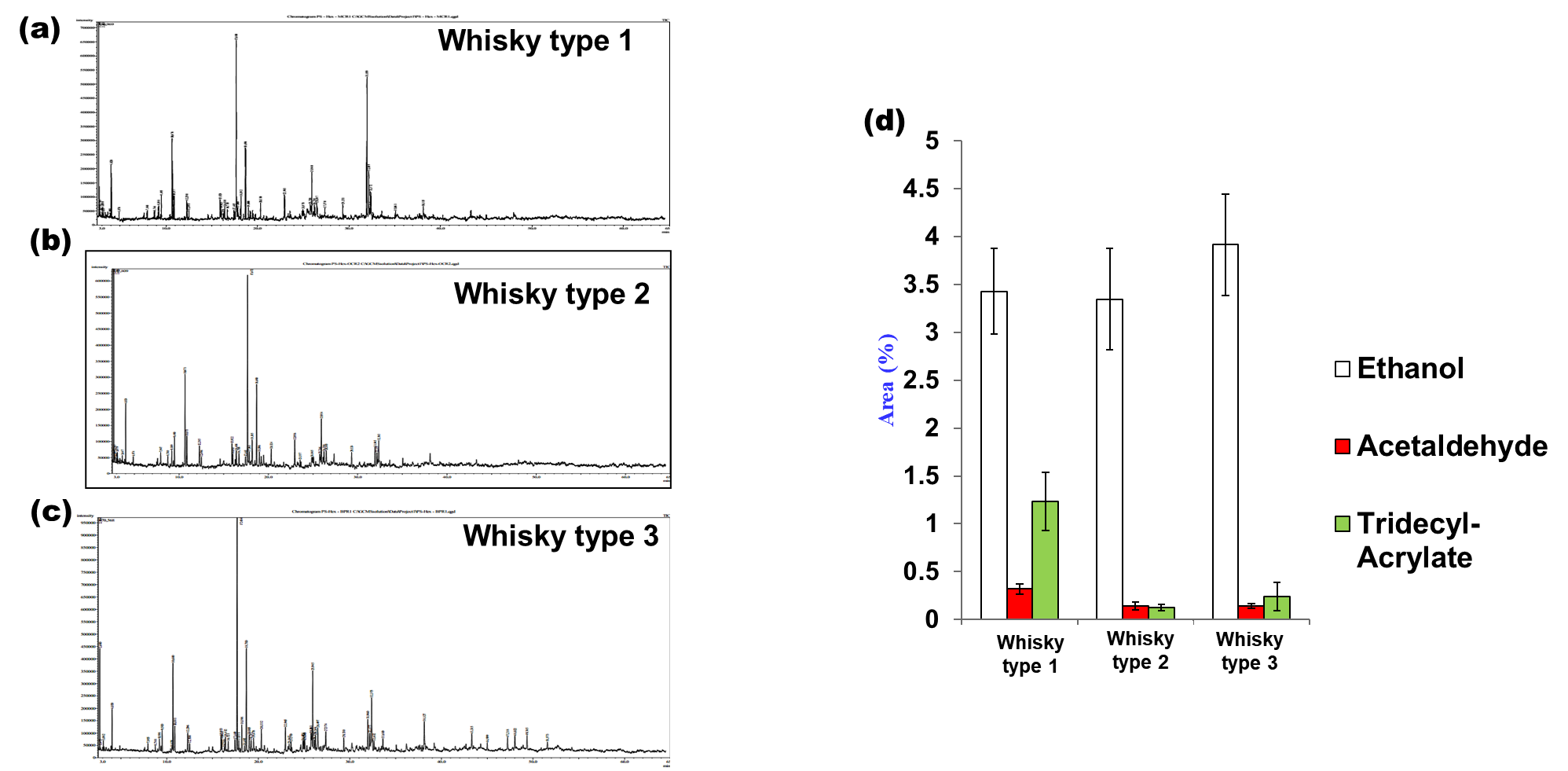


**Figure S12.** Ethanol percentage of the whisky types samples studied in the work.


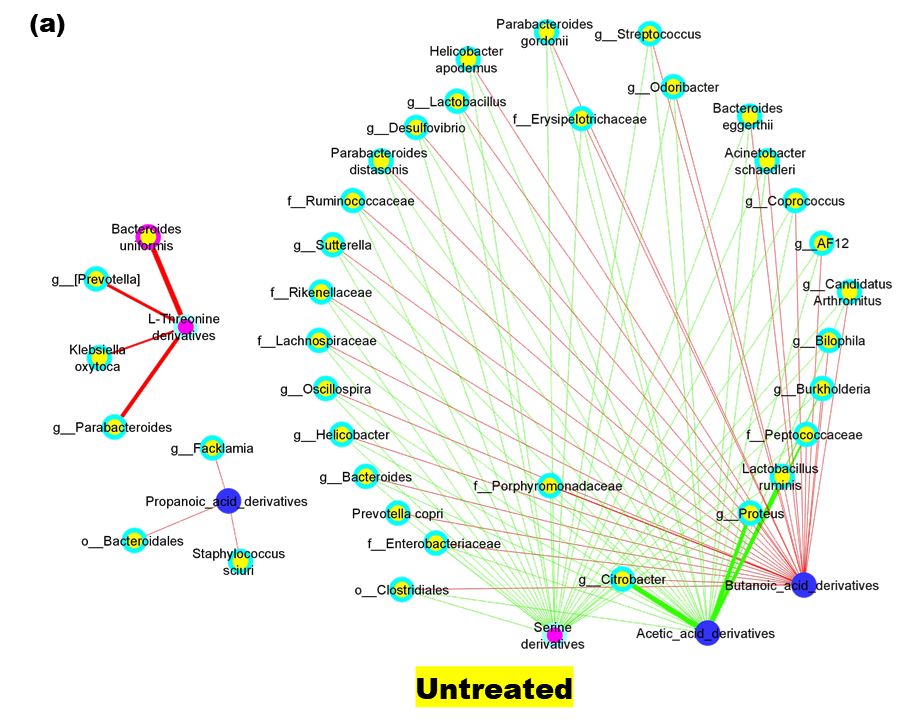


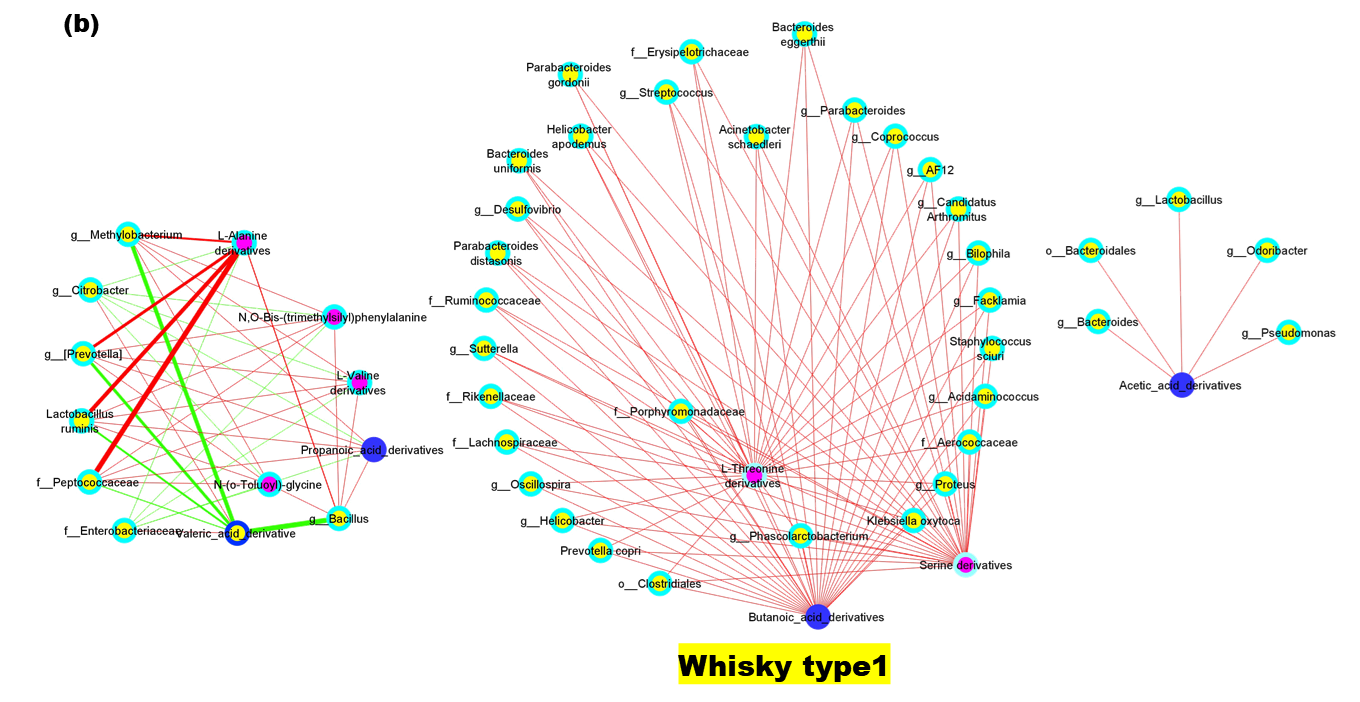


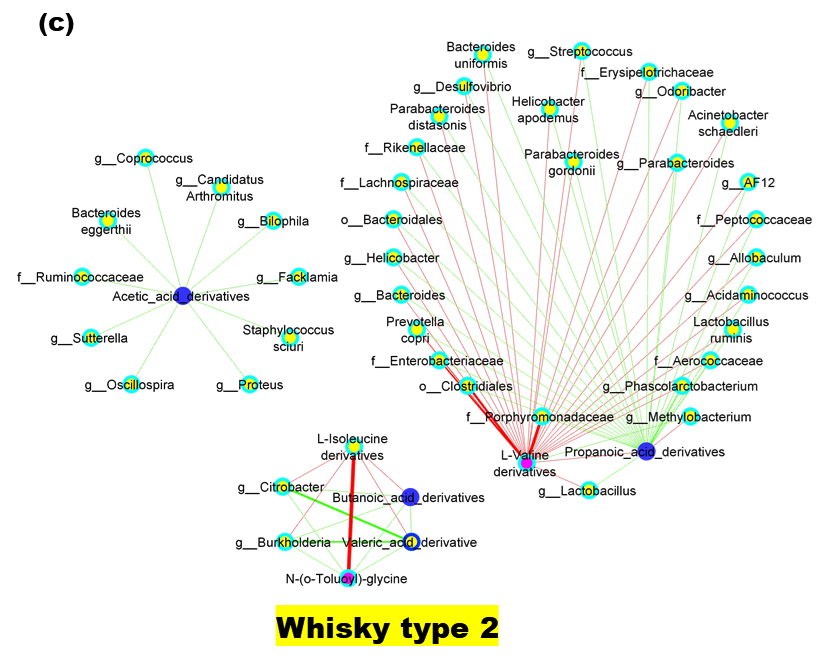


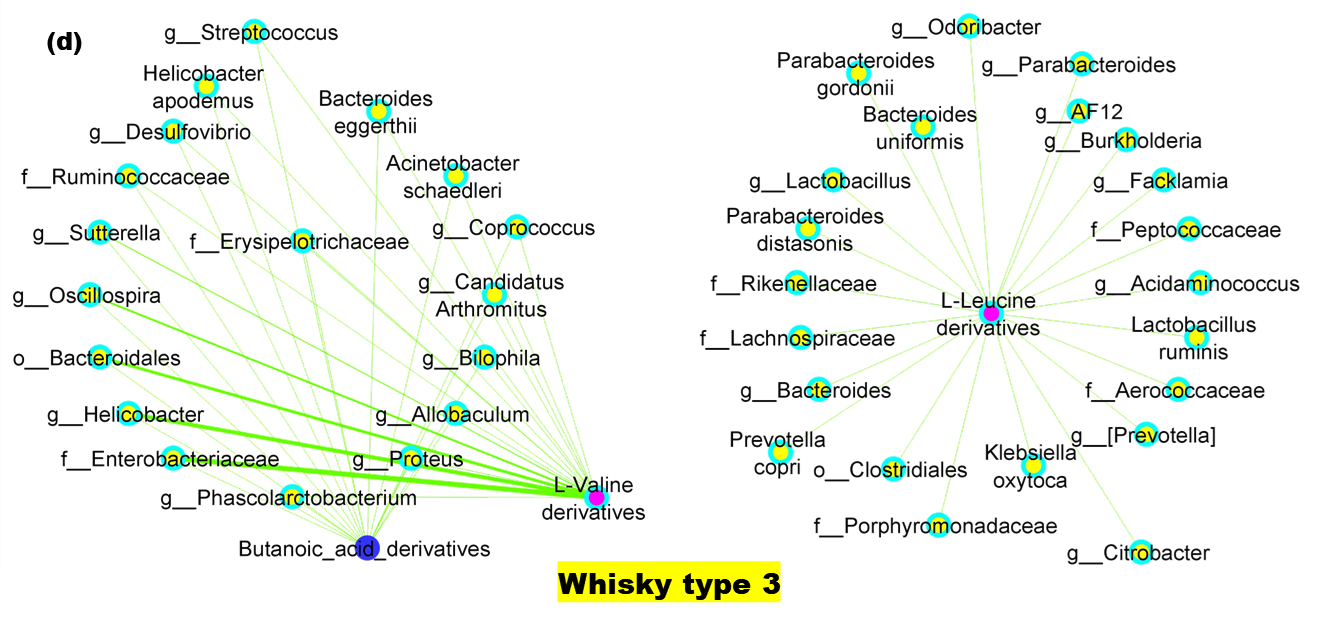


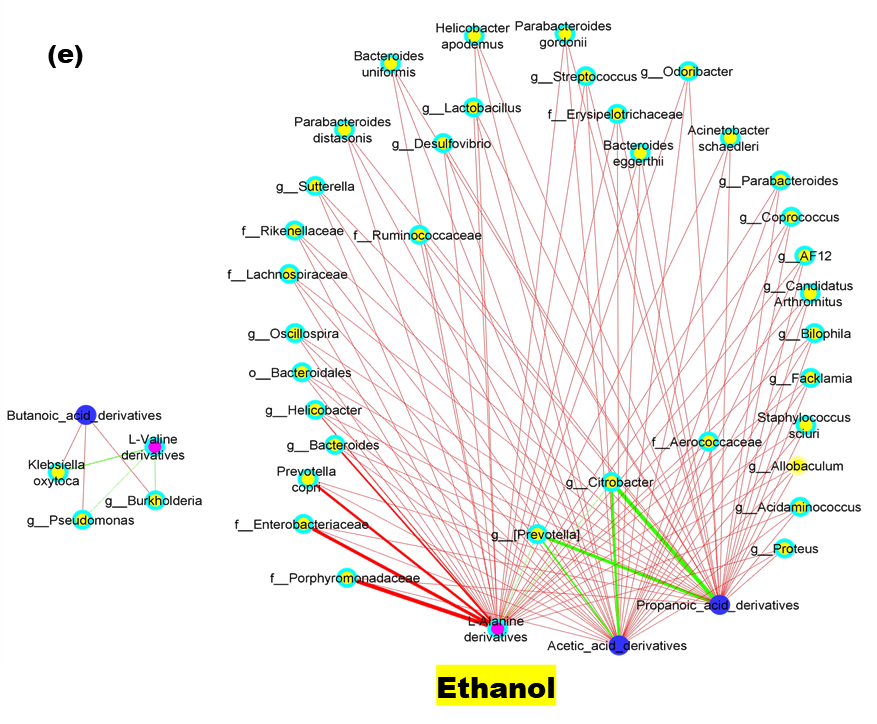


**Figure S13.** Network plot based on the correlation observed between Microbiome and metabolome in **(a)** Untreated, **(b)** Whisky type1, **(c)** Whisky type 2, **(d)** Whisky type 3 and **(e)** Ethanol treated groups {**green**-positive interactions, **red**-negative interactions, r= -1.0 to 1.0 ; *p*=0.01}**.**
