# Supplementary material for: Multi-Omics Analysis Demonstrates the Critical Role of Non-Ethanolic Components of Alcoholic Beverages in the Host Microbiome and Metabolome: A Human- and Animal-Based Study
Source: Microorganisms. 2023 Jun 5;11(6):1501. doi: 10.3390/microorganisms11061501 (PMC10301968; doi:10.3390/microorganisms11061501)
Supplement: Supplementary file 1 [file microorganisms-11-01501-s001.zip › Table S2_Partial sequences of 16S ribosomal RNA gene of uncultured bacteria submitted in NCBI.docx]

| **Table S2: Partial sequences of 16S ribosomal RNA gene of uncultured bacteria submitted in NCBI** | |
| --- | --- |
| **DGGE band no. Accession no.** | **NCBI with their accession ids** |
| Band1 | KY656572.1 |
| Band2 | KY656573.1 |
| Band3 | KY656574.1 |
| Band4 | KY656575.1 |
| Band5 | KY656576.1 |
| Band6 | KY656577.1 |
| Band7 | KY656578.1 |
| Band8 | KY656579.1 |
| Band9 | KY656580.1 |
| Band10 | KY656581.1 |
| Band11 | KY656582.1 |
| Band12 | KY656583.1 |
| Band13 | KY656584.1 |
| Band14 | KY656585.1 |
| Band15 | KY656586.1 |
| Band16 | KY656587.1 |
